# A co-delivery system based on chlorin e6-loaded ROS-sensitive polymeric prodrug with self-amplified drug release to enhance the efficacy of combination therapy for breast tumor cells

**DOI:** 10.3389/fbioe.2023.1168192

**Published:** 2023-03-29

**Authors:** Cui Wang, Xiaoqing Yang, Haibao Qiu, Kexin Huang, Qin Xu, Bin Zhou, Lulu Zhang, Man Zhou, Xiaoqing Yi

**Affiliations:** ^1^ College of Pharmacy, Gannan Medical University, Ganzhou, China; ^2^ Key Laboratory of Prevention and Treatment of Cardiovascular and Cerebrovascular Diseases, Gannan Medical University, Ganzhou, China

**Keywords:** polymeric prodrug, ROS-sensitive, synergistic chemo-photodynamic therapy, self-amplified drug release, tumor cells

## Abstract

**Background:** Recently, various combination therapies for tumors have garnered popularity because of their synergistic effects in improving therapeutic efficacy and reducing side effects. However, incomplete intracellular drug release and a single method of combining drugs are inadequate to achieve the desired therapeutic effect.

**Methods:** A reactive oxygen species (ROS)-sensitive co-delivery micelle (Ce6@PTP/DP). It was a photosensitizer and a ROS-sensitive paclitaxel (PTX) prodrug for synergistic chemo-photodynamic therapy. Micelles size and surface potential were measured. In vitro drug release, cytotoxicity and apoptosis were investigated.

**Results:** Ce6@PTP/DP prodrug micelles exhibited good colloidal stability and biocompatibility, high PTX and Ce6 loading contents of 21.7% and 7.38%, respectively. Upon light irradiation, Ce6@PTP/DP micelles endocytosed by tumor cells can generate sufficient ROS, not only leading to photodynamic therapy and the inhibition of tumor cell proliferation, but also triggering locoregional PTX release by cleaving the thioketal (TK) bridged bond between PTX and methoxyl poly (ethylene glycol). Furthermore, compared with single drug-loaded micelles, the light-triggered Ce6@PTP/DP micelles exhibited self-amplified drug release and significantly greater inhibition of HeLa cell growth.

**Conclusion:** The results support that PTX and Ce6 in Ce6@PTP/DP micelles exhibited synergistic effects on cell-growth inhibition. Thus, Ce6@PTP/DP micelles represent an alternative for realizing synergistic chemo-photodynamic therapy.

## Introduction

Recently, biodegradable and biocompatible amphiphilic polymeric micelle-based drug delivery systems have attracted much interest because of their ability to promote drug utilization and alleviate side effects of chemotherapy has played an important role (Pan et al., 2019; Zhou et al., 2020; Chen et al., 2021). Although the drug delivery systems based on polymer micelles has been extensively studied and applied in the laboratories and the clinics, further advancements are required to overcome the drawbacks of low drug-loading capacity (DLC), premature drug leakage, and insufficient drug release at the normal site. Prodrugs are derivatives of native drugs that temporarily block drug activity by modifying the active site to reduce toxicity to normal tissues, currently accounting for ∼10% drugs marketed globally. (Jubeh et al., 2020; Liu et al., 2022). Most chemotherapeutic agents exhibit poor water solubility and a short circulation lifetime owing to their innate hydrophobicity. Conversely, prodrug formulations generally exhibit good water solubility. Self-assembly of an amphiphilic polymeric prodrug can form micelles, which not only improves the dispersion and pharmacokinetic properties of chemotherapeutic drugs in aqueous conditions but also increase drug accumulation *in vivo via* the permeability enhancement and retention (EPR) effect at the lesion site (Wang K. et al., 2019; Wang H. et al., 2020; Peng et al., 2021).

The self-assembled polymeric prodrug micelles are very stable in a physiological environment and can reduce unwanted drug leakage of drugs before reaching tumor tissue, thereby effectively inhibiting side effects of chemotherapeutic drugs on normal cells (Sun et al., 2018; Shukla et al., 2022; Sun et al., 2022). The polymeric prodrug micelles not only have the advantages of improved chemical stability, high DLC and low side effects, but they can also be used as drug carriers, thereby constituting a multidrug codelivery system that can be used for combination therapy and for improving therapeutic efficacy (Li S. et al., 2020; Ma et al., 2021). Furthermore, polymeric prodrugs can be designed for exogenous (e.g., ultrasound and light) or endogenous [e.g., reactive oxygen species (ROS), reducing environment and enzymes] stimulus-responsive drug delivery to activate their toxicity and achieve controlled drug release at the tumor site (Yang et al., 2019; Ye et al., 2019; Cao et al., 2021). Previous studies have reported that the concentration of ROS in tumor cells is 10 times higher than that in normal cells owing to mitochondrial dysfunction and metabolic exuberance (Oddone et al., 2020; Palmer et al., 2020; Liang and Zhou, 2021). Therefore, the development of a ROS-sensitive prodrug micelle delivery system should facilitate the selective release of drugs in cancer cells over normal cells.

However, the concentration of ROS in cancer cells is insufficient to activate prodrug release, which is attributable to several factors, including tumor heterogeneity (Luo et al., 2019; Cao et al., 2021; Guo et al., 2021). Consequently, the frequent use of chemotherapeutic drugs can easily induce drug resistance in tumor cells (Jubeh et al., 2020; Zhang et al., 2021b; Li et al., 2021). Furthermore, single chemotherapy is inadequate for treating cancer (Wang M. et al., 2019; Zhang et al., 2021a; Pang et al., 2022). Therefore, combined chemotherapy and other treatments can effectively reduce the number of drugs used and improve treatment efficacy, such as photodynamic therapy (PDT) (Lee et al., 2021; Lv et al., 2022; Wu et al., 2022). ROS, which can induce tumor cell apoptosis or necrosis, can be produced by the reaction between photosensitizer and dissolved oxygen under light irradiation, defined as PDT. PDT is an important modality of cancer treatment owing to its advantages of minimal invasiveness and modulated phototoxicity that improves the quality of life and median survival time. (Zhou et al., 2020; Yi et al., 2021; Zhou et al., 2021; Zhou et al., 2022). PDT and chemotherapy are now emerging as new approaches to treating solid tumors. (Sun et al., 2020; Zuo et al., 2021). Therefore, ROS-sensitive multidrug polymeric prodrug delivery system can effectively kill two birds with one stone as it can not only perform PDT *via* controllable illumination, but also achieve tumor synergistic therapy by using the generated ROS to trigger the release of prodrugs for self-amplified drug release.

**SCHEME 1:**
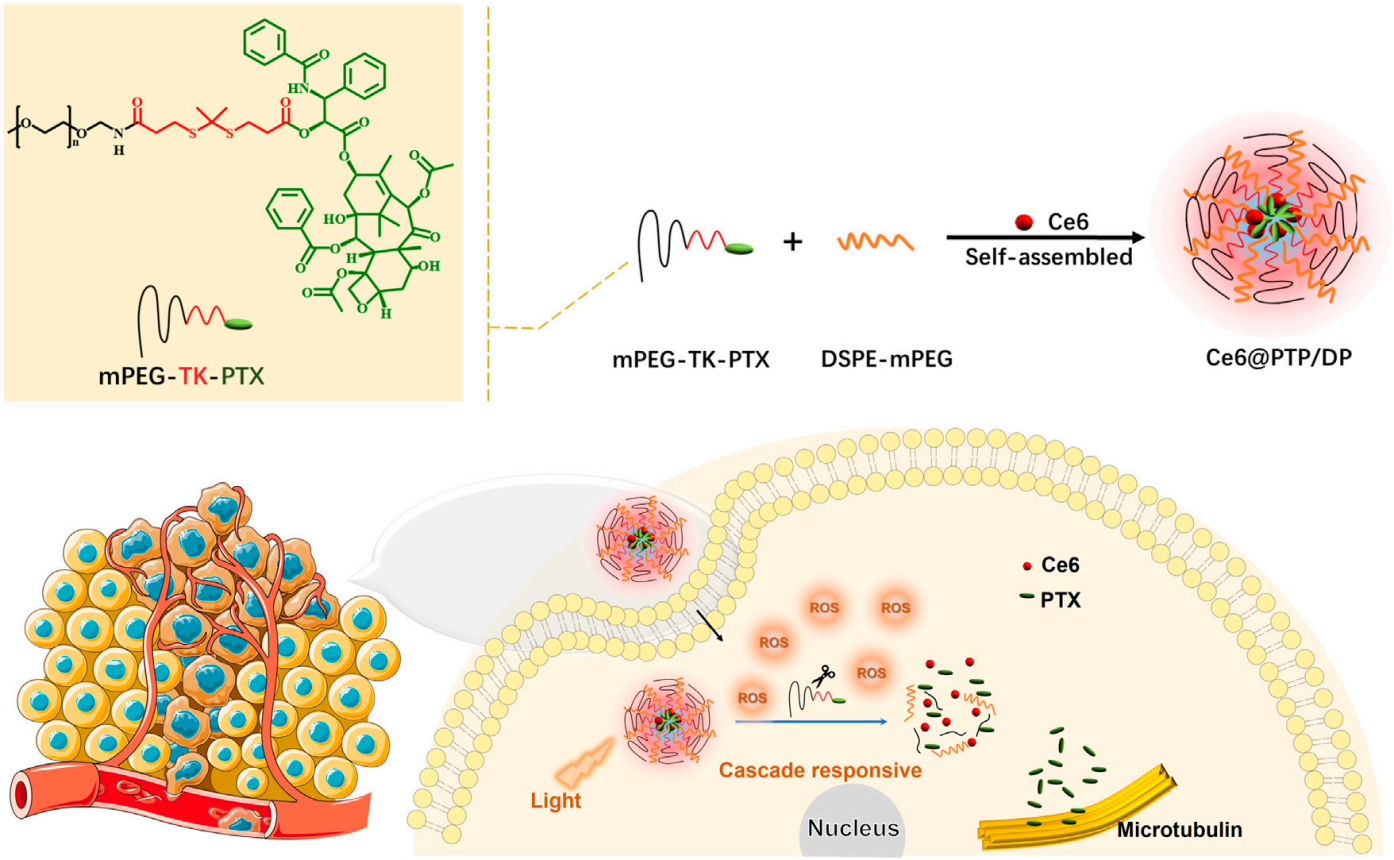
Ce6@PTP/DP self-assembly schematic diagram and its application in chemo-photodynamic therapy.

To promote the effectiveness of tumor synergistic therapy, we designed a novel ROS-sensitive polymeric prodrug micelle (Ce6@PTP/DP) with high DLC and self-amplified drug release. The multidrug delivery system comprised three parts: a ROS-sensitive polymeric prodrug methoxyl poly (ethylene glycol)-thioketal-paclitaxel (mPEG-TK-PTX, PTP), DSPE-mPEG (DP), and a traditional photosensitizer of chlorin e6 (Ce6), as illustrated in Scheme 1. The ROS-sensitive polymeric multidrug delivery system has the following advantages: 1) stability in blood circulation, excellent biocompatibility and high DLC (21.7% for PTX and 7.38% for Ce6); 2) PTX release from polymeric prodrug micelles can be effectively triggered under oxidative conditions or light irradiation by breaking the TK bond; 3) the hydrodynamic size of Ce6@PTP/DP micelles was 80.4 nm, which was stable and easily phagocytized by tumor cell; and 4) synergistic therapeutic efficacy of chemotherapy-PDT in HeLa cells.

## Materials and methods

### Materials

DSPE-mPEG (Mw:2000) and paclitaxel (PTX) were purchased from Xi’an Ruixi Biotechnology Technology Co., Ltd. (Xi’an, China). Methyl sulfoxide (DMSO), suberic acid, N, N′-dicyclohexylcarbodiimide (DCC), 4-dimethylaminopyridine (DMAP), 3-(4,5-dimethylthiazol-2-yl)-2,5-diphenyl tetrazolium bromide (MTT) was obtained from MACKLIN reagent (Shanghai, China). Dichloromethane, petroleum ether, ethyl acetate and the other reagents were analytical or HPLC grade, obtained from Guangdong Guanghua Technology Co., Ltd. (Guangdong, China).

### Cell culture

Human cervical cancer carcinoma HeLa cells were cultured in high-glucose DMEM containing 10% fetal bovine serum (FBS) and maintained in a humid environment atmosphere of 5% CO_2_ at 37°C.

### Characterizations

^1^H NMR spectra (400 MHz, CDCl_3_) were obtained by a spectrometer (Bruker AV-400, USA). Micelle’s size, size distribution in aqueous solution were determined by dynamic light scattering (DLS, ZS90, Malvern). The morphology of the micelles was investigated by means of transmission electron microscopy (FEI Tecnai 12).

### Synthesis of Thioketal (TK)

Briefly, 3-mercaptopropionic acid (5.31 g, 50 mmol) and anhydrous acetone (5.81 g, 100 mmol) were dissolved in dry hydrogen chloride and stirred for 3 h at room temperature. After that, when the color of the solution turns orange, it was put back to cold conditions for recrystallization. Then washed several times and the product was filtered with cold petroleum ether and water. Finally, the product was dried under a vacuum (Pei et al., 2018; Liu et al., 2022).

### Synthesis of PTX-TK-COOH

The dry PTX (200.00 mg, 2.34 mmol), HOOC-TK-COOH (59.10 mg, 2.34 mmol), DMAP (2.86 mg, 0.234 mmol) were dissolved in 2 mL dry THF to the flask. DCC (531.10 mg, 2.57 mmol) was added slowly dropwise and condensed at 40°C with reflux stirring for 48 h under argon atmosphere. The reaction products were washed with ethyl acetate, then collected the filtrate to evaporated under reduced pressure. Then the crude product was obtained by filtration with ether. Finally, the crude product was purified by column chromatography (ethyl acetate: acetic acid = 100:1).

### Synthesis of mPEG-TK-PTX (PTP)

NHS (22.89 mg, 0.20 mmol), EDC (19.69 mg, 0.20 mmol), PTX-TK-COOH (200.00 mg, 0.20 mmol) were dissolved in dry DMF and activated by stirring for 3 h. Then mPEG-NH_2_ (200.00 mg, 0.20 mmol) was added and dialyzed after 48 h. The product was purified by HPLC (acetonitrile and water = 1:1).

### Preparation of PTP, Ce6@DP and Ce6@PTP/DP Micelles

Typically, PTP (10 mg) was dissolved in DMSO (1 mL) and the mixture was added dropwise to ultrapure water (9 mL) under vigorous stirring. Then continued stirring for 30 min. Next, the solution was transferred to a dialysis bag (Mw:1000) and dialyze overnight to obtain PTP micelles. The same treatment noted that the loading photosensitizer needed to be protected from light: 1) Ce6 loaded DSPE-mPEG, called Ce6@DP. 2) Ce6 loaded mPEG-TK-PTX and DSPE-mPEG, named Ce6@PTP/DP. The concentration of PTX and Ce6 in each micelle was calculated by HPLC and UV-Vis.

### Characterization of micelles morphology

The polymer micelles were observed using transmission electron microscopy (TEM). The samples were prepared as follows: the sample micelles of aqueous solution (0.5 mg/mL) were dropped on a copper grid and stained with 0.2% (w/v) phosphotungstic acid solution to observe the micelle morphology.

### Measurement of micelles size and distribution and stability

The micelles size and distribution were determined by DLS at 25°C, 173°, and 633 nm with a helium-neon laser source. The concentration of PTP, Ce6@DP, and Ce6@PTP/DP were 1 mg/mL.

### Release of PTX from Ce6@PTP/DP micelles

The release of PTX from PTP and Ce6@PTP/DP was measured by HPLC. The measurement was performed as follows: PTX release from PTP and Ce6@PTP/DP was measured by dialysis, 1 mL of freshly prepared PTP and Ce6@PTP/DP micelles solution was transferred into a dialysis bag (Mw: 1000) and then placed into 20 mL of release medium in aqueous solution and gently shaken at 37°C for 150 rpm. 1 mL of release solution was removed at a pre-set time and then supplemented with an equal amount of fresh release medium.

### Hydrolysis reactions of Ce6@PTP/DP

The hydrolysis reaction of Ce6@PTP/DP was monitored by incubation in 10 mM H_2_O_2_ and white light (100 mW cm^-2^) under different conditions of the hydrodynamic diameter as well as the distribution of Ce6@PTP/DP micelles.

### Detection of ROS in solution

ABDA was used to test the generation of ROS. Typically, 15 μL of ABDA solution (4.5 mg/mL, DMSO) were added to the micelle of Ce6@PTP/DP, then the mixed solution (1.5 mL) were put in a small beaker and irradiated with white light (100 mW/cm^−2^). Finally, the absorbance values were recorded every 1 min of irradiation to monitor the decomposition process of ABDA. The absorbance of ABDA was recorded and obtained the decay rate of the photsensitization process under white light (100 mW/cm^‐2^).

### Intracellular ROS generation study at the cellular level

Treated centration of intracellular ROS was detected using 2ʹ,7ʹ-dichlorofluorescein diacetate (DCFH-DA) as a probe. The HeLa cells were seeded in 6-well plates and cultured for 24 h. The cells were incubated with PTP, Ce6@DP and Ce6@PTP/DP for 4 h, respectively. That the cells untreated were the control group. After treatment, the cells were washed three times with PBS. Then 10 μM DCFH-DA was added to replace the medium at 37°C for 30 min, followed by white light (100 mW/cm^−2^, 10 min). Finally, the cells were cleaned three times with PBS, and then the fluorescence of DCFH-DA in the cells was detected with CLSM.

### Cell uptake

HeLa cells were inoculated in cell culture dishes and incubated for 24 h Ce6@DP and Ce6@PTP/DP micelles were then added separately and incubated for another 4 h. The above cells were washed three times with PBS using Hoechst 33258, and then incubated for 20 min. Finally, the cells were further washed three times with pre-chilled PBS for CLSM observation.

### Cytotoxicity assay

*In vitro* cytotoxicity of all drug forms on cancer HeLa cells was assayed by MTT. HeLa cells were inoculated on 96-well plates at a density of 5 × 10^3^ cells per well and cultured overnight. First, the cells were treated with different concentrations of PTX, PTP, Ce6@DP and Ce6@PTP/DP. After 48 h of incubation, 10 µL of MTT (5 mg/mL) was added to each well and further incubated for 4 h. Finally, the absorbance of each well at 490 nm was measured by microplate readout. In another experiment, the cells were incubated with PTX, PTP, Ce6@DP and Ce6@PTP/DP for 4 h, followed by irradiation at white light (100 mW/cm^−2^, 10 min). After further incubation for 48 h, cell viability was determined by MTT method.

### Apoptosis detection

Annexin V-FITC and PI were used to assess the cell viability of the HeLa cells. The cells were cultured overnight in 6-well plates at 2.0 × 10^5^ cells per well. Then, the cells were incubated with PTP, Ce6@DP and Ce6@PTP/DP (PTX or Ce6 equivalent dose 5 μg/mL and 1.7 μg/mL) for 24 h. After 15 min of reaction at room temperature according to the instructions of the Annexin V-FITC Apoptosis Assay Kit. Finally, the samples mixed and placed on the ice were detected by flow cytometry (BD FACS Canto Ⅱ, USA) within 1 h.

### Hemolysis assays

The hemocompatibility of PTP, Ce6@DP and Ce6@PTP/DP at different concentrations were studied by hemolysis assay. Freshly mice blood was diluted by PBS and centrifuged to collect red blood cells (RBCs). 2% RBC suspension was used for hemolysis study. Then, these micelles were added and mixed by vortex and incubated at 37°C for 6 h. Then mixtures were centrifuged at 1200 rpm for 10 min. The supernatant was collected and amount of hemoglobin released was recorded on an enzyme Markers at 510 nm and 540 nm. Double-distilled water was used as positive control and PBS was used as negative control.

The hemolysis ratio of RBCs was calculated according to the following formula: Hemolysis (%) = (*A*_sample_–*A*_negative_)/(*A*_positive_–*A*_negative_) × 100%, where, *A*_sample_ was the absorbance of sample, *A*_negative_ was the absorbance of negative control and *A*_positive_ was the absorbance of positive control. All hemolysis experiments were carried out in triplicate.

### Statistical analysis and data availability

All values in the text are described as mean ± standard deviation (SD) and were analyzed using GraphPadPrism 5.01 software. Statistical analysis was performed using one-way ANOVA followed by Dunnett’s comparison test. *p* < 0.05 is different (*), *p* < 0.01 is significantly different (**), *p* < 0.001 is significantly different (***).

## Results and discussion

### Synthesis of TK, PTX-TK-COOH, mPEG-TK-PTX (PTP)

**FIGURE 1:**
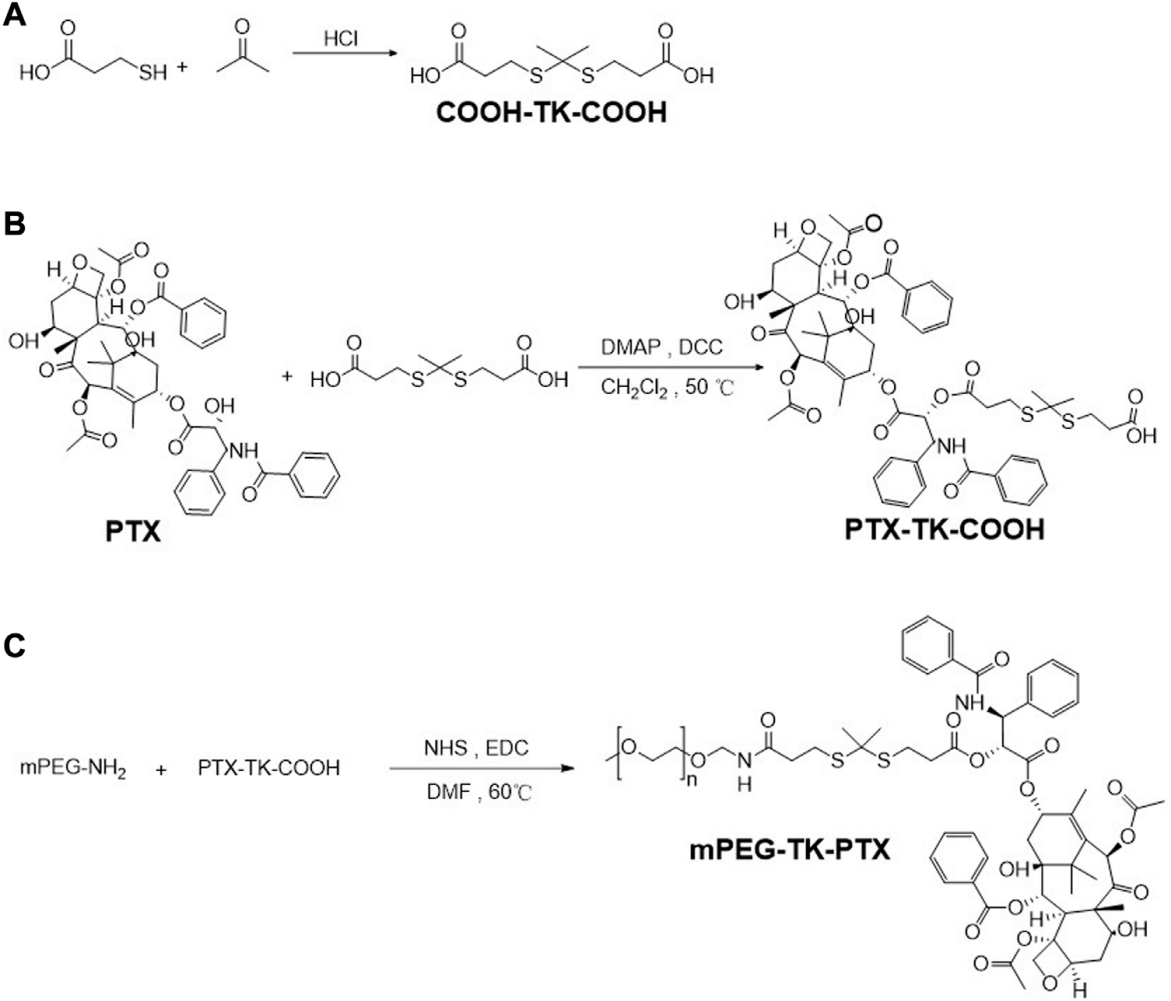
The synthesis routs of TK **(A)**, PTX-TK-COOH **(B)**, and mPEG-TK-PTX **(C)**, respectively.

**FIGURE 2:**
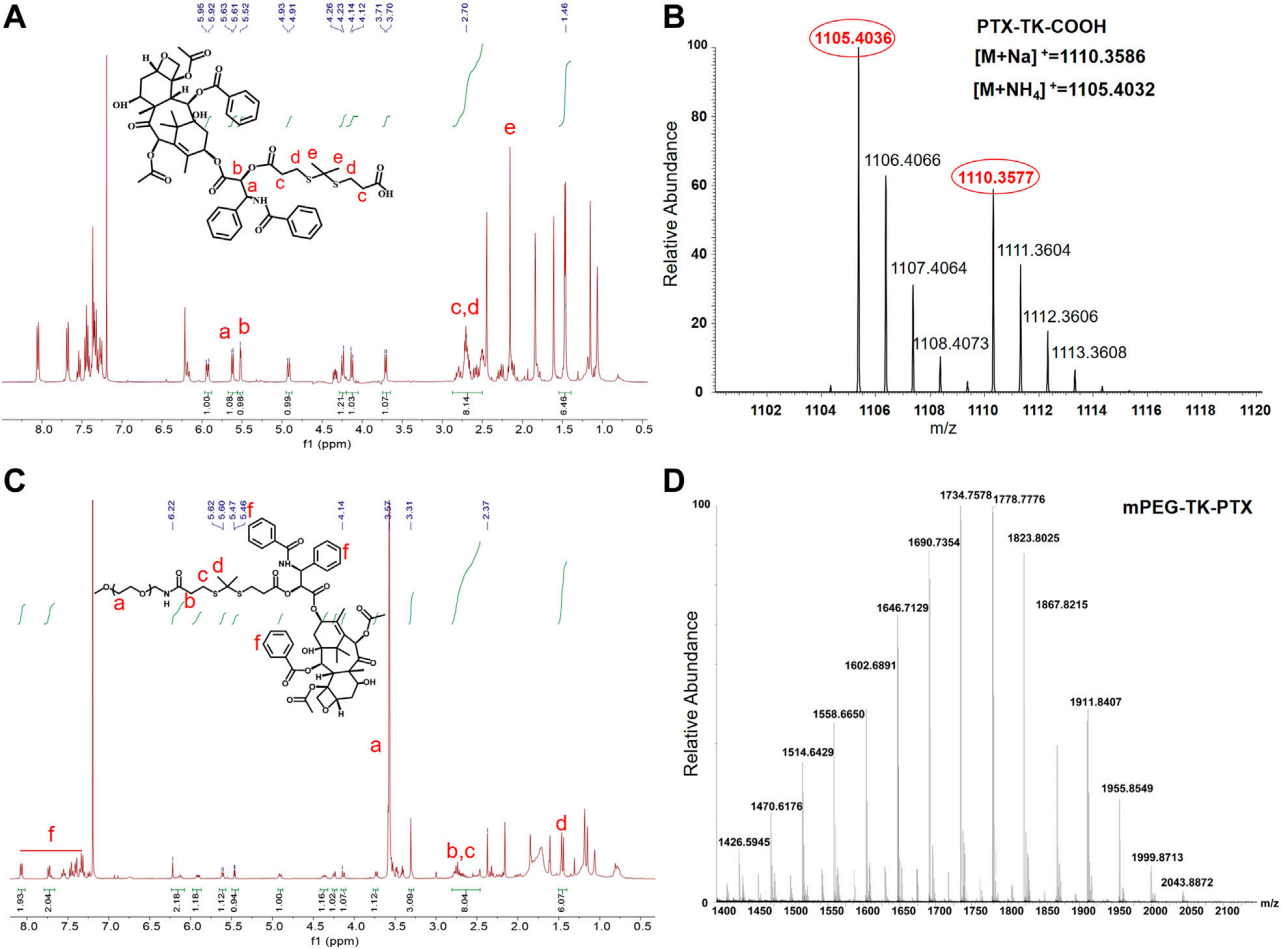
**(A)** ^1^H NMR spectrum (400 MHz, CDCl_3_) of PTX-TK-COOH. **(B)** Mass spectrum (MS) of PTX-TK-COOH. **(C)** ^1^H NMR spectrum (400 MHz, CDCl_3_) of mPEG-TK-PTX. **(D)** MS of mPEG-TK-PTX.

To obtain the ROS-sensitive polymeric prodrug micelle (Ce6@PTP/DP), Ce6 with excellent capacity for PDT and ROS-sensitive PTX prodrug for enhanced chemotherapy-PDT were utilized. The polymeric prodrug PTP was synthesized, and its detailed synthesis is presented in Figure 1. First, the ROS-sensitive linker of TK was synthesized, and the ^1^H NMR spectrum of TK was consistent with that reported in previous reports (Supplementary Figure S1) (Li Y. et al., 2020; Xu et al., 2020; Zhang et al., 2022). Then, the ROS-sensitive prodrug PTX-TK-COOH was obtained *via* a condensation reaction between PTX (^1^H NMR result shown in Supplementary Figure S2) and TK, and the chemical framework of PTX-TK-COOH was clarified using the ^1^H NMR result (Figure 2A) which revealed that the peak of the 2′-hydroxyl group of PTX at 3.6 ppm vanished and the signal of 2′-CH shifted from 4.7 to 5.9 ppm (Cao et al., 2018; Chang et al., 2020). Furthermore, the signals at *δ* 1.52 and *δ* 5.52 were attributed to methyl protons belonging to the TK structure and methyne proton belonging to the C2′(CH)-OH of PTX, respectively. Moreover, the appearance of the mass spectrum peak at 1105.4036 ([M + NH_4_]^+^, calculated 1105.4032) and 1110.3577 ([M + Na]^+^, calculated 1110.3586) confirmed that PTX-TK-COOH was successfully synthesized and that the esterification reaction between PTX and TK occurred at the 2′-hydroxyl group of PTX (Figure 2B). Then, the ROS-sensitive polymeric prodrug of PTP was synthesized by reacting mPEG-NH_2_ (^1^H NMR result shown in Supplementary Figure S3) with PTX-TK-COOH. The characteristic signals of PEG, TK, and PTX at *δ* 3.57, 2.5–2.8, and 7.31–8.10, respectively, appeared in the spectrum of PTP (Figure 2C), indicating its successful preparation. Furthermore, the matrix-assisted laser desorption/ionization time of flight mass spectrometry spectrum revealed that the molecular weight of PTP was 1734.8 Da (Figure 2D). These findings imply that the ROS-sensitive amphiphilic polymer prodrug PTP was successfully synthesized.

### Preparation PTP, Ce6@DP and Ce6@PTP/DP micelles

**FIGURE 3:**
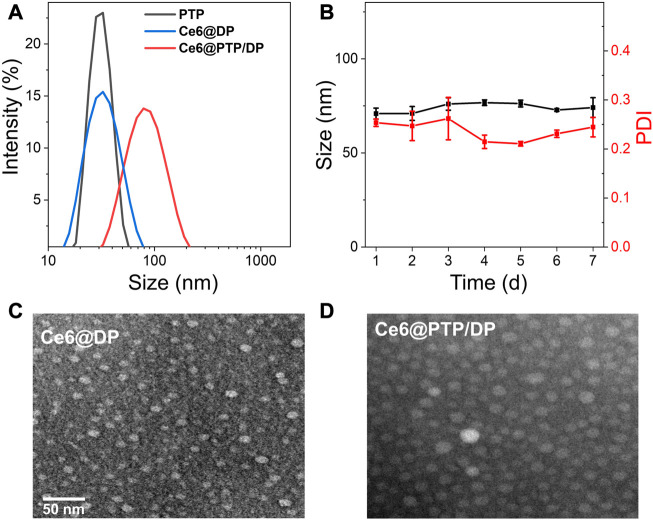
**(A)** Size distribution PTP, Ce6@DP and Ce6@PTP/DP. **(B)** Seven-day size change of Ce6@PTP/DP. Data represent the mean ± s.d (n = 3). **(C, D)** TEM images of Ce6@DP and Ce6@PTP/DP. Scale bar: 50 nm.

The ROS-sensitive multifunction polymeric micelles with the function of self-amplified drug release comprising Ce6, PTP and DP, were termed Ce6@PTP/DP. The control micelles without the chemotherapeutic drug PTX comprised Ce6 and DP, and were referred to as Ce6@DP. Only PTP constituted another control micelle without the function of ROS generation. The above micelles were acquired *via* dialysis in aqueous solution. The characterizations of PTP, Ce6@DP and Ce6@PTP/DP micelles are shown in Figure 3A; Table 1. The hydrodynamic sizes of PTP, Ce6@DP and Ce6@PTP/DP micelles were 30.9 ± 1.3 nm (PDI = 0.057 ± 0.038), 29.4 ± 1.0 nm (PDI = 0.134 ± 0.053), and 80.4 ± 2.2 nm (PDI = 0.217 ± 0.006), respectively.

**TABLE 1:** Properties of the PTP, Ce6@DP and Ce6@PTP/DP micelles.

Samples	DLC (wt%)		Mass ration (PTX: Ce6)	Size (nm)	PDI
	PTX	Ce6			
PTP	46.9	/^a^	/^a^	30.9 ± 1.3	0.057 ± 0.038
Ce6@DP	/^b^	6.42	/^b^	29.4 ± 1.0	0.134 ± 0.053
Ce6@PTP/DP	21.7	7.38	2.94	80.4 ± 2.2	0.217 ± 0.006

^a^ No Ce6 was encapsulated in the PTP, micelles.

^b^ No PTX, was encapsulated in the Ce6@DP, micelles.

### Stability evaluation of the micelles

The stability of the Ce6@PTP/DP micelles for synergistic chemo-photodynamic therapy was monitored at room temperature for 1 week. The hydrodynamic size of Ce6@DP and Ce6@PTP/DP micelles did not significantly change during a week (Supplementary Figure S4; Figure 3B), indicating that the Ce6@PTP/DP micelles with PEG shell exhibited good stability in an aqueous environment, and their adequate size enabled their enrichment at the tumour site owing to improved enhanced permeability and retention (EPR) effect. Furthermore, transmission electron miscroscopy (TEM) images confirmed that the Ce6@DP and Ce6@PTP/DP micelles exhibited obvious spherical boundaries (Figures 3C,D), with sizes of ∼10 and 20 nm, respectively. The smaller size measured *via* TEM compared with that measured *via* dynamic light scattering (DLS) results can be primarily attributed to the shrinkage of the PEG shell upon drying. The absorption peak of the Ce6@PTP/DP micelles in aqueous solution at 402 nm was mainly attributed to the characteristic absorption of Ce6, and a strong fluorescence peak was observed at 684 nm (Supplementary Figures S5, S6). The DLC of Ce6 in the Ce6@DP and Ce6@PTP/DP micelles were determined using the standard curve of UV-Vis absorption of Ce6, which were 6.42% and 7.38%, respectively (Table 1; Supplementary Figure S7). The PTP and Ce6@PTP/DP micelles exhibited high DLC for PTX, reaching up to 46.9% and 21.7%, respectively, which were significantly higher than that of conventional nanomedicine (usually ≤10%, w/w). These results indicate that the prepared polymeric prodrug micelles have good stability and excellent DLC for synergistic chemo-photodynamic therapy.

### ROS production in solution

The production of ROS under light irradiation was analyzed in an aqueous solution using the ROS indicator, 9,10-anthracenediylbis (methylene)dimalonic acid (ABDA) *via* UV-Vis spectrophotometer. ABDA effectively merges with ROS, lowering its characteristic UV-Vis absorption peaks at 378 and 400 nm. Therefore, the reduction in the characteristic peak absorption intensity of ABDA is equivalent to the amount of ROS produced under light irradiation. The characteristic peaks at 378 and 400 nm of the anthracene moiety in ABDA reduced with increasing of irradiation duration (Supplementary Figure S8). After 5.5 min of light irradiation, the absorption intensity at 400 nm declined by ≥ 40%. When the illumination time was extended to 10.5 min, the absorption intensity decreased by ∼60%, thereby confirming that the polymeric prodrug micelles stacked with the photosensitizer Ce6 exhibited satisfactory ROS production under light irradiation for PDT.

### *In vitro* release of PTX from PTP and Ce6@PTP/DP micelles

**FIGURE 4:**
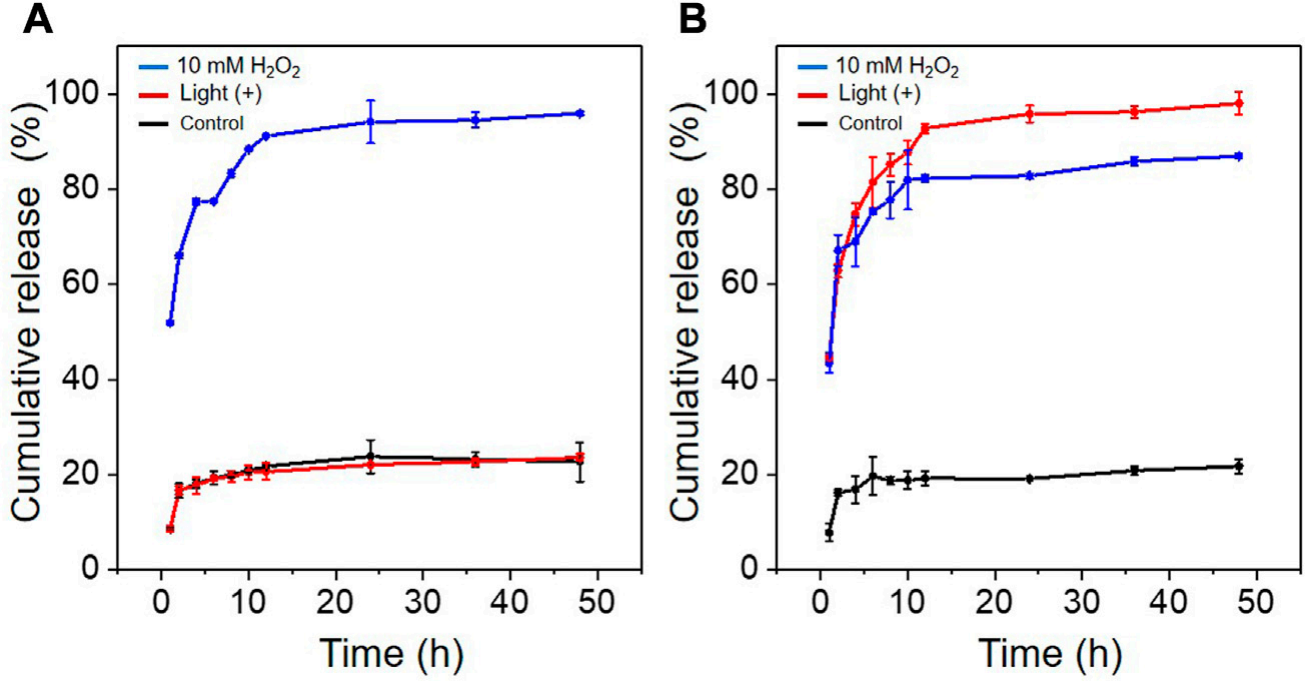
**(A)** Cumulative release of PTX from PTP under different conditions. **(B)** Cumulative release of PTX from Ce6@PTP/DP under different conditions: control (without light and H_2_O_2_); white light, 100 mW/cm^‐2^, 10 min; 10 mM H_2_O_2_.

The effects of different environments on the *in vitro* release of PTX from PTP and Ce6@PTP/DP micelles were investigated under the condition of 10 mM H_2_O_2_ and light irradiation by HPLC. As shown in Figures 4A, B, When PTP and Ce6@PTP/DP micelles were not treated by light irradiation or H_2_O_2_, the release of PTX was greatly inhibited, 21.74% and 22.62% of PTX was released within 48 h, which indicated that the polymeric prodrug micelles had good stability and sufficient protection ability for drug leakage of PTX. However, the release of PTX increased considerably under 10 mM H_2_O_2_ and light stimulation, as can be seen in the PTP micelles with 23.6% and 95.9% release within 48 h, respectively. However, Ce6@PTP/DP micelles released 86.9% and 98.0% within 48 h. This indicates that the release capacity of PTX can be stimulated under oxidative conditions, which helps to achieve the controlled release of drugs and improve the therapeutic efficiency. The reason why the release of PTX can be triggered under oxidative conditions is mainly attributed to the fact that the TK functional group is oxidized and the link between PTX and PEG is cut off. The above shows that light irradiation can not only perform PDT, but also trigger the release of PTX to achieve self-amplified release of drug.

### Light-triggered changes of the Ce6@PTP/DP micelles

**FIGURE 5:**
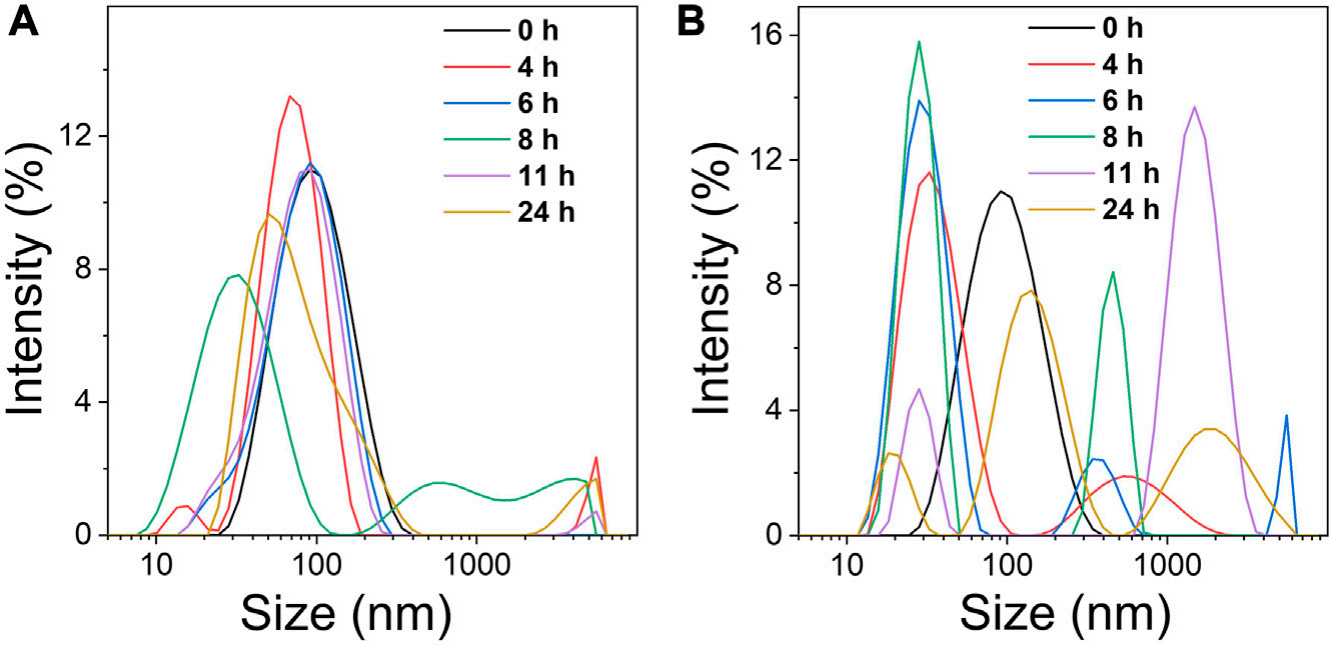
**(A)** Changes in the micelle size distribution of Ce6@PTP/DP at different times under 10 mM H_2_O_2_. **(B)** Changes in the micelle size distribution of Ce6@PTP/DP at different times under white light (100 mW/cm^−2^), respectively.

TK, a sulfhydryl-based ROS-sensitive functional group, has often been used for ROS-sensitive drug delivery (Wang D. et al., 2020). The introduction of TK between PEG and PTX to prepare the prodrug PTP, induces a cascade reaction which cleaves PTP upon ROS production. Thus, the ROS produced *via* PDT induces the degradation of the Ce6@PTP/DP micelles to facilitate PTX release. Consequently, DLS was used to assess the behavior of ROS production of the Ce6@PTP/DP micelles. The change in the size of the Ce6@PTP/DP micelles in response to H_2_O_2_ and light irradiation was monitored over time. The Ce6@PTP/DP micelles exhibited significant size change at a H_2_O_2_ concentration of 10 mM (Figure 5A). After the micelles were infused with H_2_O_2_ for 4 h, the maximum size of the multiple peaks increased up to ∼5000 nm, probably owing to the oxidation of the TK group by H_2_O_2_, which influences to the separation of hydrophilic and hydrophobic chain segments of the Ce6@PTP/DP micelles, leading to the breakdown of the micellar structure. Furthermore, the change in size of the Ce6@PTP/DP micelles in response to light irradiation was identified *via* DLS (Figure 5B). As ROS produced *via* PDT under light irradiation can influence the degradation of the TK group, thereby disrupting the self-assembly of the Ce6@PTP/DP micelles, as presumed, the signal of DLS becomes more complex. For example, after 4 h of light irradiation, a new signal peak of ∼800 nm appears. Furthermore, a new signal peak at ∼5000 nm appears after 6 h of light irradiation. These findings indicate that the prepared Ce6@PTP/DP micelles undergo oxidation in response to H_2_O_2_ and light irradiation, suggesting that ROS produced *via* PDT can influence the degradation of micelles.

### ROS production of the prepared micelles *in vivo*

**FIGURE 6:**
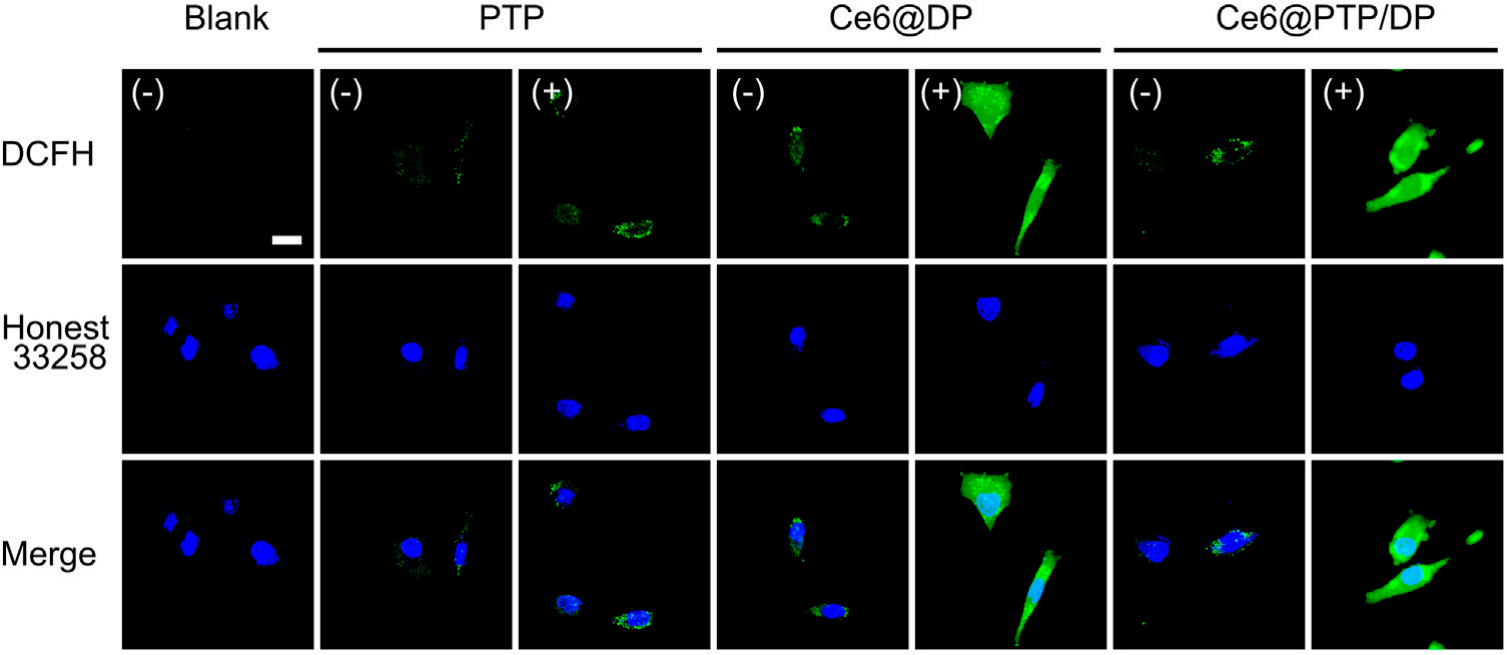
ROS generation studies of PTP, Ce6@DP and Ce6@PTP/DP micelles in HeLa cells. Using DCFH-DA as general ROS fluorescence indicators (green) and the nuclei were stained by Hoechst 33258 (blue), respectively.

Efficient drug uptake by tumor cells is a recommended feature of prepared micelles. The cellular uptake of the Ce6@PTP/DP and Ce6@DP micelles was observed *via* confocal laser scanning microscopy (CLSM) in HeLa cells. Bright fluorescence signals for the Ce6@PTP/DP and Ce6@DP micelles were observed in the cytoplasm *via* CLSM, whereas almost no fluorescence signals were observed in the nuclear region (Supplementary Figure S9). This may be because of the fact that the diameter of the nuclear pore complex is 10 nm, and the size of the Ce6@PTP/DP and Ce6@DP micelles is much larger than that, which is why fluorescence signals of the nano-micelle is hardly detected in the cell nucleus area. CLSM was used to determine the ROS production ability of the PTP, Ce6@DP and Ce6@PTP/DP micelles under white light irradiation using 2′, 7-dichlorofluorescein acetate (DCFH-DA) as a fluorescence probe. The intrinsic fluorescence intensity of DCFH-DA is very low, however, it can be oxidized to DCF with strong green fluorescence in the presence of ROS. Therefore, the ability of the prepared polymeric micelles to produce ROS can be indirectly demonstrated by monitoring the fluorescence intensity of intracellular DCF. When the cells were treated with DCFH-DA only in the absence of light, there was some green fluorescence in the cells. This was because intracellular enzymes or natural antioxidants scavenge these oxygen radicals in normal cells. The fluorescence signals detected *via* CLSM after all the micelles were incubated with HeLa cells were very weak without light irradiation, suggesting that the proportion of ROS produced was very small, and insufficient to oxidize the ROS fluorescent probe DCFH-DA (Figure 6). However, the green fluorescence signals of the Ce6@DP and Ce6@PTP micelles were incubated in HeLa cells under light irradiation were markedly improved, thereby signifying that the polymeric micelles loaded with Ce6 produced a large proportion of ROS and could oxidize DCFH-DA. However, unlike the Ce6@DP andCe6@PTP/DP micelles, the fluorescence signals of the PTP micelles in HeLa cells were very weaker following light irradiation. These results indicate that the Ce6@PTP/DP micelles exhibit satisfactory ROS production in cells and can be further employed for combination therapy.

### Hemolysis test

**FIGURE 7:**
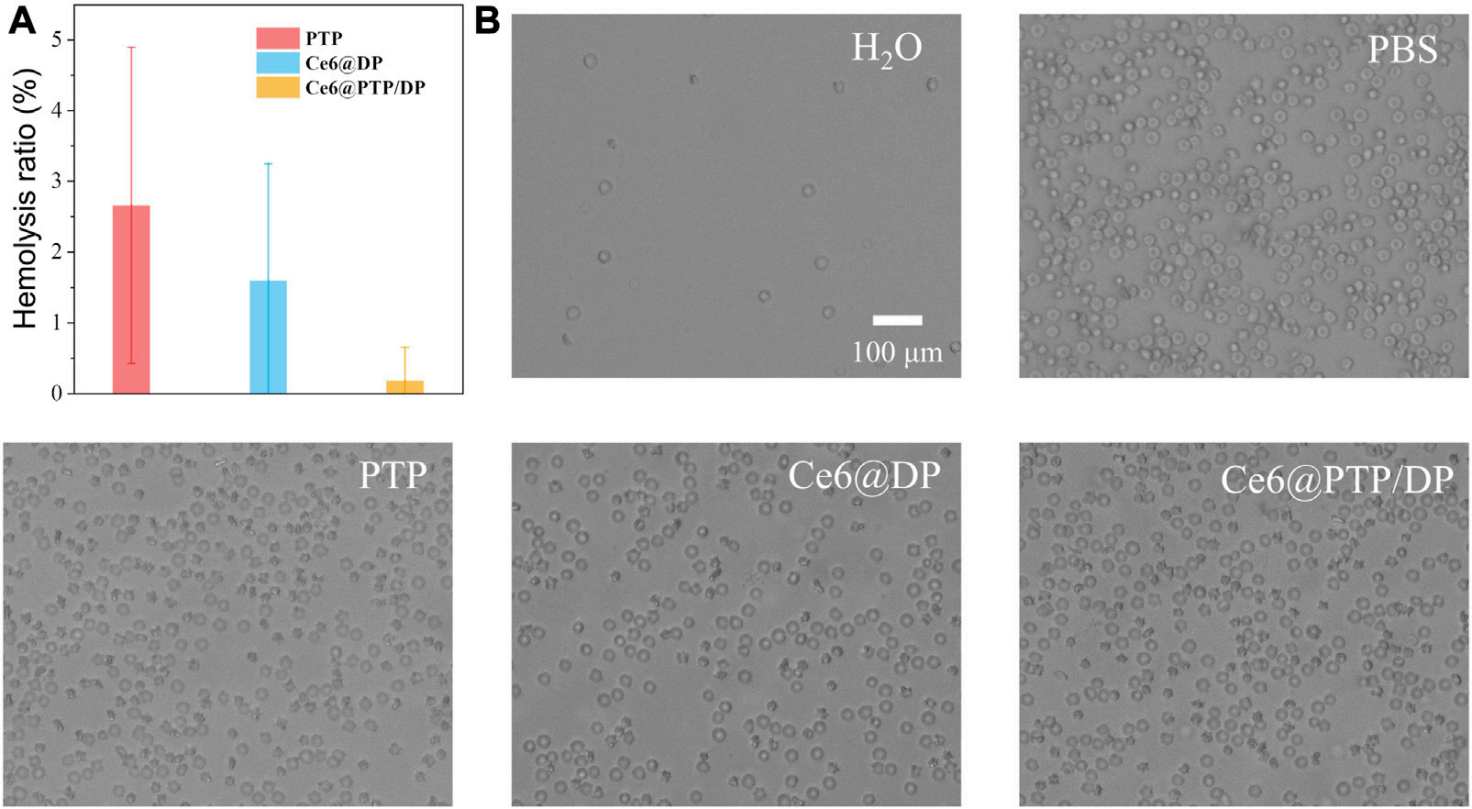
**(A)**The hemolysis ratio induced by PTP, Ce6@DP, Ce6@PTP/DP micelles incubated at 37°C for 6 h in dark. Data represent the mean ± s.d (n = 3). **(B)** Optical microscopic observation of the dispersion states of the erythrocytes after incubated with H_2_O, PBS, PTP, Ce6@DP, Ce6@PTP/DP micelles for 6 h. Scale bar: 100 μm.

Good blood compatibility is a prerequisite of polymeric prodrug micelles for clinical application. The PTP, Ce6@DP and Ce6@PTP/DP were incubated with red blood cell solution at 37°C for 6 h in the dark, and the rate of hemolysis was determined *via* UV-Vis measurement of the hemoglobin released from damaged red blood cells (Figure 7). Generally, the lower the rate of hemolysis, better the blood biocompatibility of polymeric prodrug micelles, and these micelles can be intravenously administered when the hemolysis rate is ≤ 10%. Almost no heme was found to be released into the supernatant after the PTP, Ce6@DP and Ce6@PTP/DP micelles were incubated with the red blood cells (Supplementary Figure S10). Furthermore, the hemolysis rate of the PTP, Ce6@DP and Ce6@PTP/DP micelles was <5%, morphology of the red blood cells was intact after following incubation, and proportion of red blood cells was equivalent to phosphate-buffered saline. These findings suggest that the Ce6@PTP/DP micelles have good blood biocompatibility, and can be delivered to the lesion site in a passively targeted manner *via* intravenous administration.

### *In vitro* cytotoxicity assay

**FIGURE 8:**
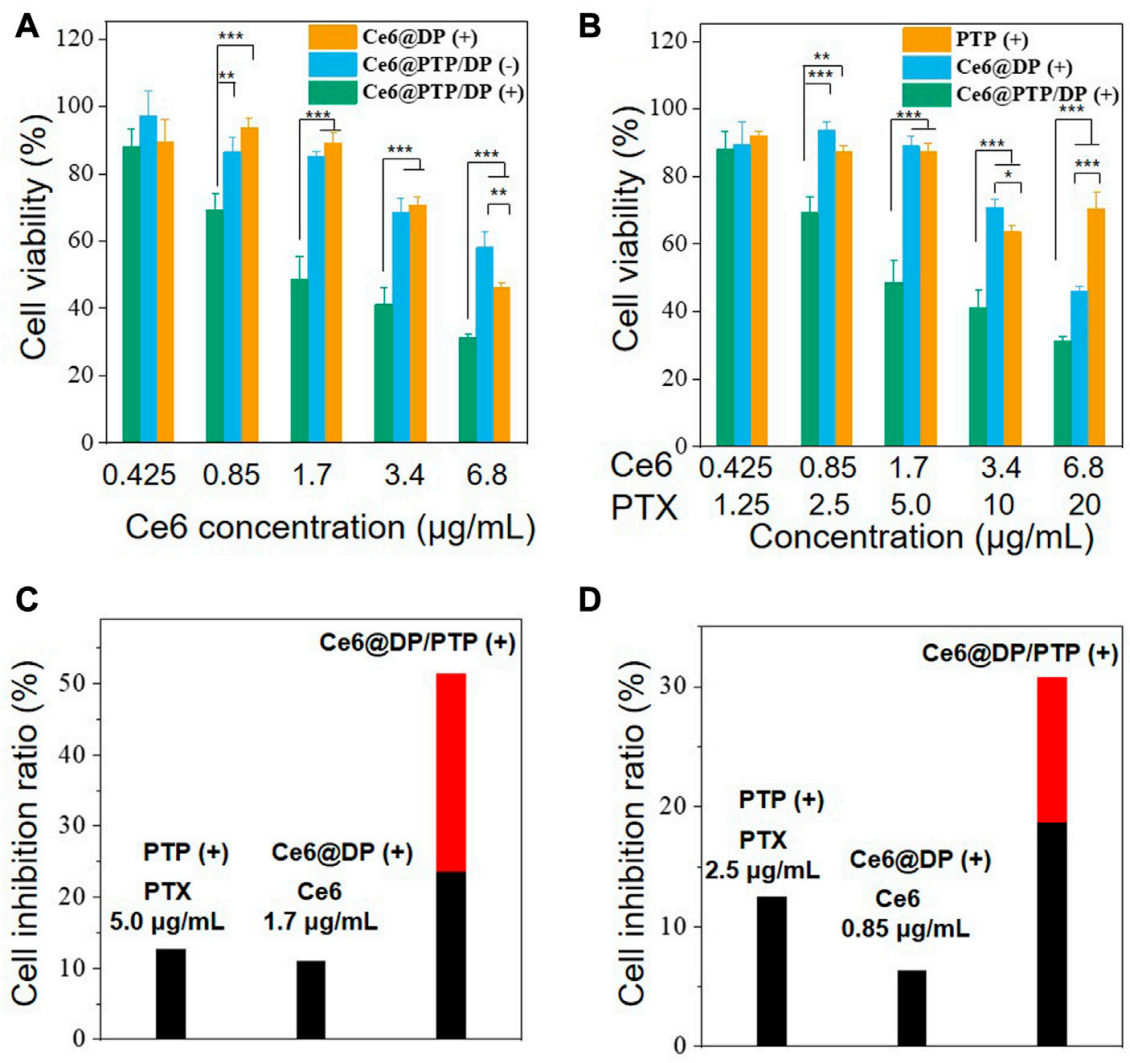
**(A)** MTT assay of Free PTX, PTP, and PTP (+) (white light, 100 mW cm^−2^, 10 min) in HeLa cells after incubation for 48 h **(B)** MTT assay of Ce6@PTP/DP, Ce6@DP (+) and Ce6@PTP/DP (+) (white light, 100 mW cm^−2^, 10 min) in HeLa cells after incubation for 48 h. Data represent the mean ± s.d (n = 3), *p < 0.05, **p < 0.01, ***p < 0.001, respectively. **(C, D)** The inhibition ratios of PTP (+), Ce6@DP (+), Ce6@PTP/DP (+) micelles treated cells upon light (white light, 100 mW cm^−2^, 10 min). The red bar denotes the additional cell inhibition ratio gained whenCe6@PTP/DP micelles upon light irradiation are combined, compared with the sum of PTP, Ce6@DP micelles upon light irradiation (white light, 100 mW cm^−2^, 10 min), respectively.

To investigate the synergistic effect of the PTX prodrug and Ce6 photosensitizer, an MTT assay was performed to determine the cytotoxicity of free PTX, and the PTP, Ce6@DP, and Ce6@PTP/DP micelles in HeLa cells. Compared with the condition of without light irradiation, cell viability did not reduce following light irradiation, and cell viability was >90%, implying that the HeLa cell activity was not hindered following light irradiation for 10 min (white light, 100 mW cm^−2^) (Supplementary Figure S11). Furthermore, the cytotoxicity of free PTX, PTP (−) (without light irradiation), and PTP (+) (white light, 100 mW cm^−2^, 10 min) was dose-dependent. Compared with the PTP (−) and PTP (+) groups, free PTX demonstrated more inhibition, probably owing to the extended release of PTX from PTP (Supplementary Figure S12). There was no substantial difference between the effect of PTP (−) and PTP (+) on HeLa cell proliferation signifying that irradiation did not affect the cytotoxicity of the PTP micelles. Subsequently, for the Ce6@DP micelles, Ce6@DP (−) exhibited minimal effects on cell proliferation, with a concentration of Ce6 up to 6.8 μg mL^-1^, whereas Ce6@DP (+) effectively hinders tumor cell growth, implying that the ROS produced by Ce6@DP under light irradiation can effectively kill tumor cells and play a role in PDT (Supplementary Figure S13; Figure 8A). For the Ce6@PTP/DP micelles, their inhibitory effect was augmented by light irradiation, comparable with that of the Ce6@DP micelles (Figure 8A). The cytotoxicity of the PTP, Ce6@DP, and Ce6@PTP/DP micelles in HeLa cells under light irradiation was further used to assess the synergistic effect of chemo-photodynamic therapy. Ce6@PTP/DP (+) demonstrated enhanced cytotoxicity compared with that of PTP (+) and Ce6@DP (+) (Figure 8B). This result indicates that the codelivery system comprising the photosensitizer and chemotherapeutic prodrug can effectively merge chemo-photodynamic therapy to achieve synergistic treatment. Furthermore, the chemo-photodynamic therapy group of Ce6@PTP/DP (+) produced a higher cell inhibition ratio than that of the sum of the chemotherapy group of PTP (+) and the PDT group of Ce6@DP (+) at the concentrations of PTX and Ce6 of 2.63 and 1.42 μg mL^−1^, respectively (red bar represents the increased cell inhibition ratio) (Figure 8C). The findings of the cell inhibition ratio was comparable with that of PTP (+), Ce6@PTP (+), and Ce6@PTP/DP (+) with the concentrations of PTX and Ce6 of 2.5 and 0.85 μg mL^−1^, respectively, in HeLa cells (Figure 8D). Thus, these results indicate that the ROS-sensitive cascaded multi-drug delivery system Ce6@PTP/DP micelles exhibit great potential for synergistic chemo-photodynamic therapy.

**FIGURE 9:**
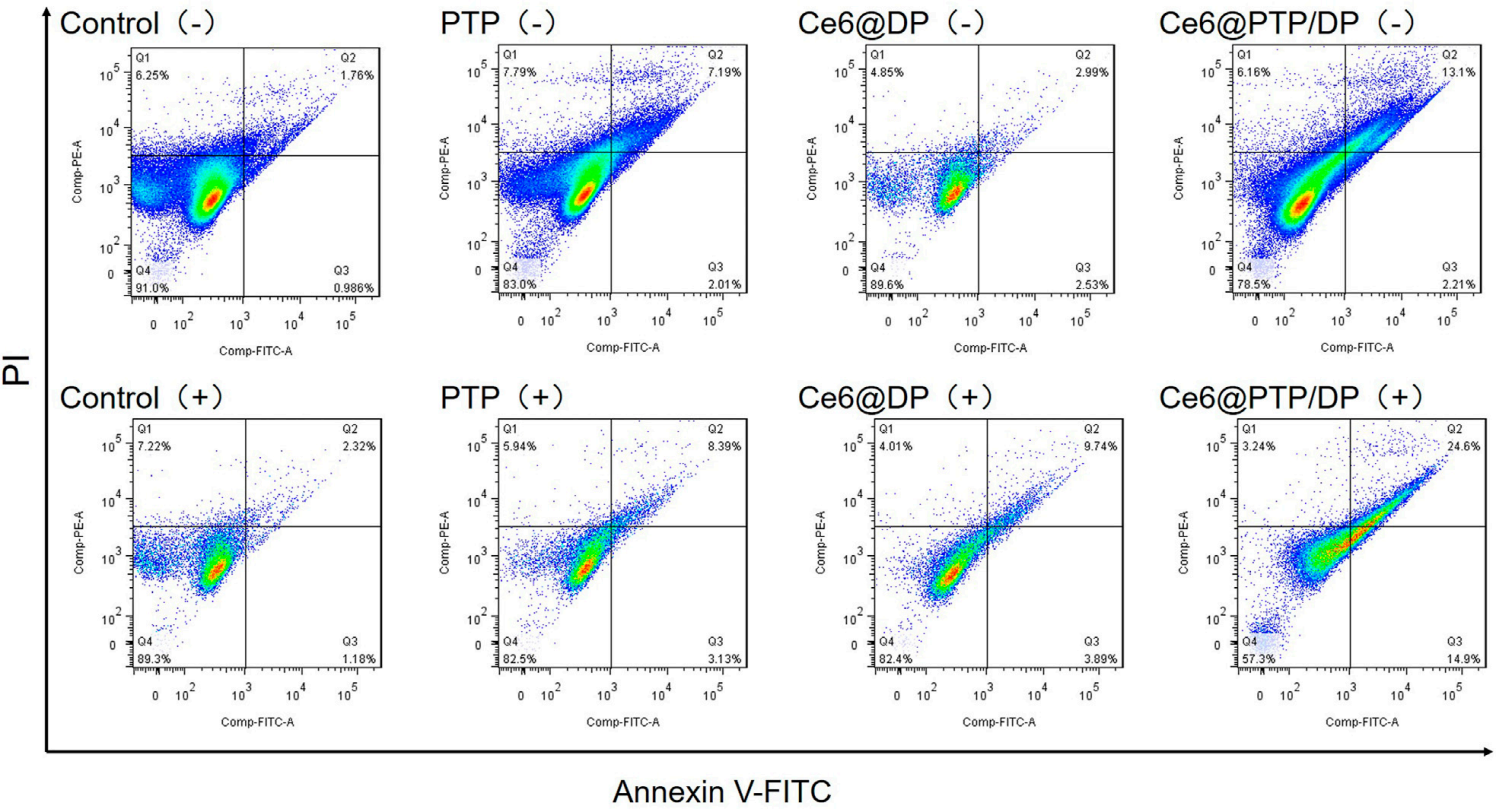
Apoptosis of HeLa cells induced by PTP, Ce6@DP, Ce6@PTP/DP for 48 h (−) (without light irradiation), and (+) (white light, 100 mW cm^−2^, 10 min).

Subsequently, the ability of micelles to induce apoptosis in HeLa cells under various treatments was evaluated using Annexin V-FITC/PI. Apoptosis was calculated as the percentage of early and late apoptosis. As was shown in Figure 9, control (−) and control (+) were 2.75% and 3.50%, which expressed the similar rate before and after light exposure. Apoptosis increased to 9.20% and 11.52% for the chemotherapy group of PTP (−) and PTP (+) respectively compared to the control group. The release of PTX from PTP is thought to be responsible for the inhibition observed. Then the PDT group of Ce6@DP (−) and Ce6@DP (+) were 5.52% and 13.62%. We demonstrate that apoptosis of cells can occur in PDT. But the chemo-PDT group of Ce6@PTP/DP (−) and Ce6@PTP/DP (+) showed increasingly grow from 15.31% to 39.30%. We also acknowledge that the rate of apoptosis in the Ce6@PTP/DP group under light condition was higher than that in any of the PDT and chemotherapy group. Finally, examination results illustrate that PTP, Ce6@DP and Ce6@PTP/DP micelles have the same trend in apoptosis and cytotoxicity, which verifies our results from another view that the ROS-sensitive cascaded co-delivery system Ce6@PTP/DP micelles have amplify the release of chemotherapy drugs potential in synergistic chemotherapy-phototherapy.

## Conclusion

In conclusion, we designed a polymeric prodrug co-delivery system, named Ce6@PTP/DP, which comprises ROS-sensitive PTX and Ce6 photosensitizer for the synergistic chemo-PDT against tumor cells. The Ce6@PTP/DP micelles produced a considerable amount of ROS with under light irradiation, which stimulated PDT, inhibited tumor cell proliferation, and triggered PTX release from the prodrug PTP, thereby improving its therapeutic effecacy. The Ce6@PTP/DP micelles exhibited good size uniformity in aqueous conditions; good stability and biocompatibility, satisfactory DLC for PTX and Ce6, which can be easily taken up by tumor cells; and excellent ROS production under light irradiation, which influences PTX release. Moreover, light irradiated Ce6@PTP/DP micelles significantly inhibited HeLa cell growth compared with single drug-loaded micelles of PTP and Ce6@DP. Thus, this ROS-sensitive cascaded multi-drug delivery system demonstrated a synergistic effect on HeLa cell growth inhibition. Overall, our findings demonstrated a facile strategy to develop ROS-sensitive co-delivery micelles with a cascade reaction to stimulate the adequate release of chemotherapeutic drugs that for synergistic chemo-photodynamic therapy.

## Data Availability

The original contributions presented in the study are included in the article/Supplementary Material, further inquiries can be directed to the corresponding authors.
