# Supplementary material for: A co-delivery system based on chlorin e6-loaded ROS-sensitive polymeric prodrug with self-amplified drug release to enhance the efficacy of combination therapy for breast tumor cells
Source: Front Bioeng Biotechnol. 2023 Mar 29;11:1168192. doi: 10.3389/fbioe.2023.1168192 (PMC10090272; doi:10.3389/fbioe.2023.1168192)
Supplement: Supplementary file 1 [file DataSheet1.docx]

Supplementary Material

**A co-delivery system based on a ROS-sensitive polymeric prodrug with self-amplified drug release for enhancing combination therapy**

*Cui Wang,*^a^ *Xiaoqing Yang,*^a^ *Haibao Qiu,*^a^ *Kexin Huang,*^a^ *Qin Xu,*^a^ *Bin Zhou,*^a^ *Lulu Zhang,*^a^ *Man Zhou*,*^,a^ *Xiaoqing Yi*,*^,a,b^

*^a^* College of Pharmacy, Gannan Medical University, Ganzhou 341000, China.

*^b^* Key Laboratory of Prevention and Treatment of Cardiovascular and Cerebrovascular Diseases, Gannan Medical University, Ganzhou 341000, China

* Corresponding author. E-mail: baiyuwawa-zhouman@163.com. (M. Zhou)

keyi0115@126.com (X. Yi).


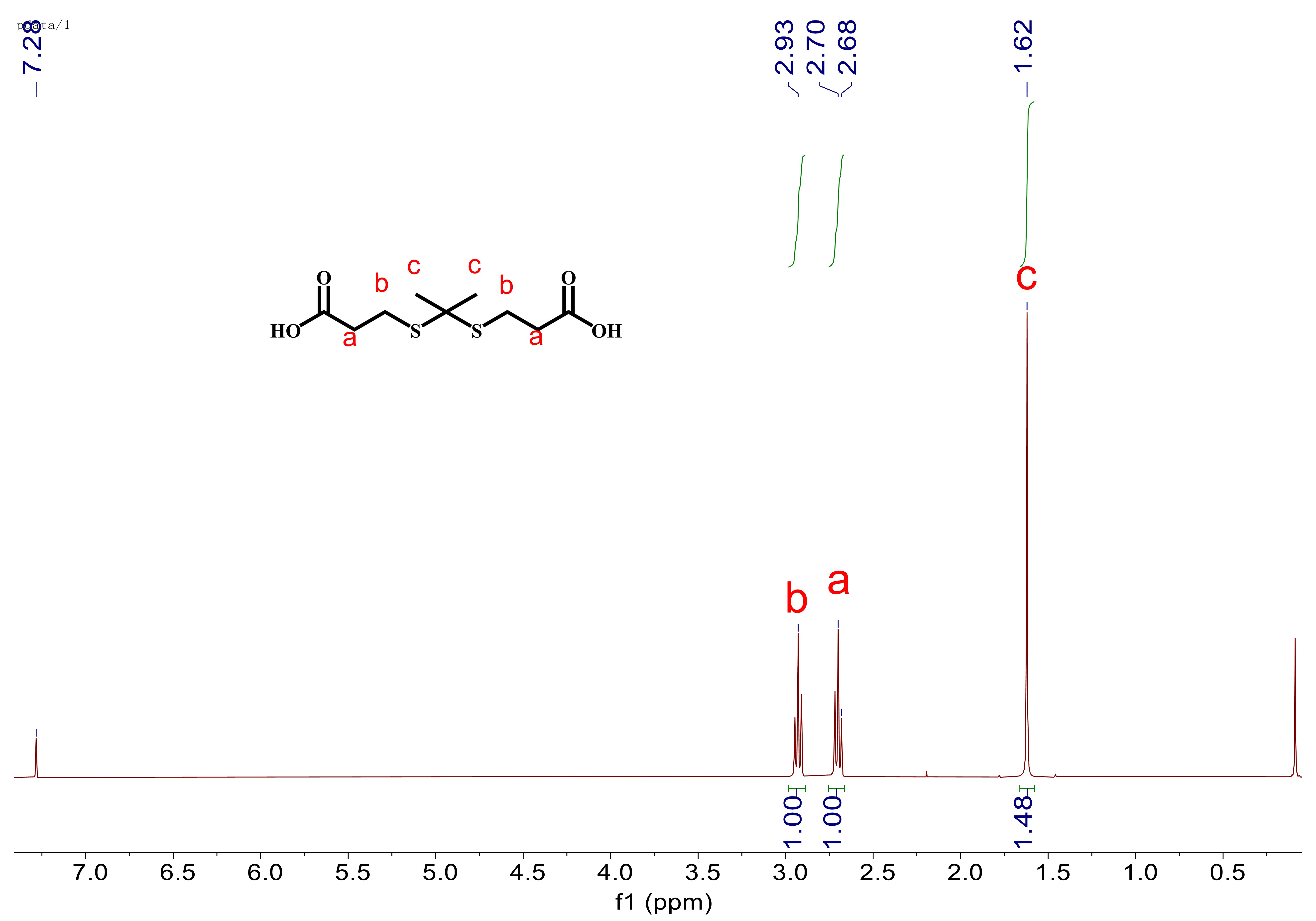


**Figure S1.** Structure and ^1^H NMR spectrum (400 MHz, CDCl_3_) of TK.


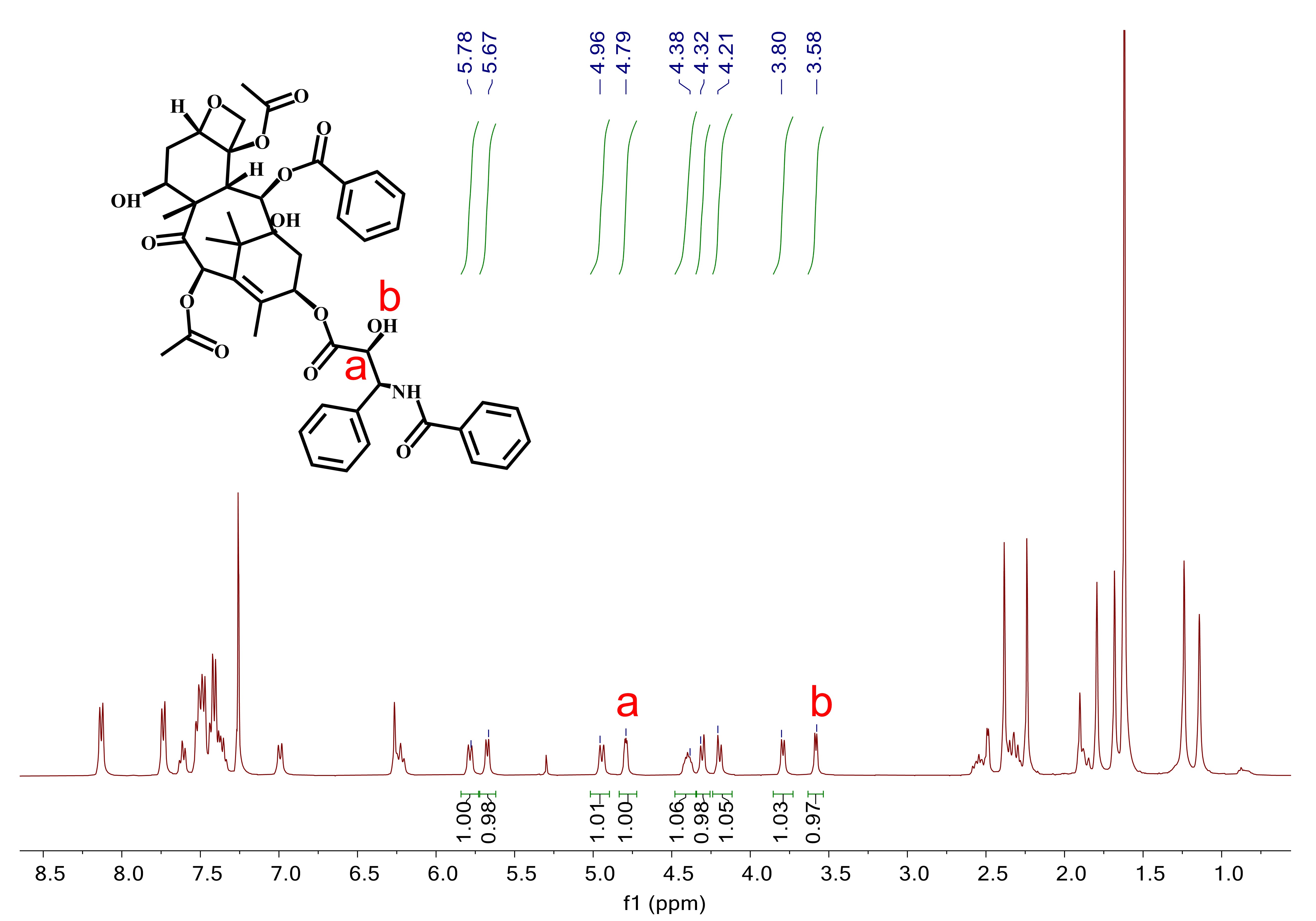


**Figure S2.** Structure and ^1^H NMR spectrum (400 MHz, CDCl_3_) of PTX.


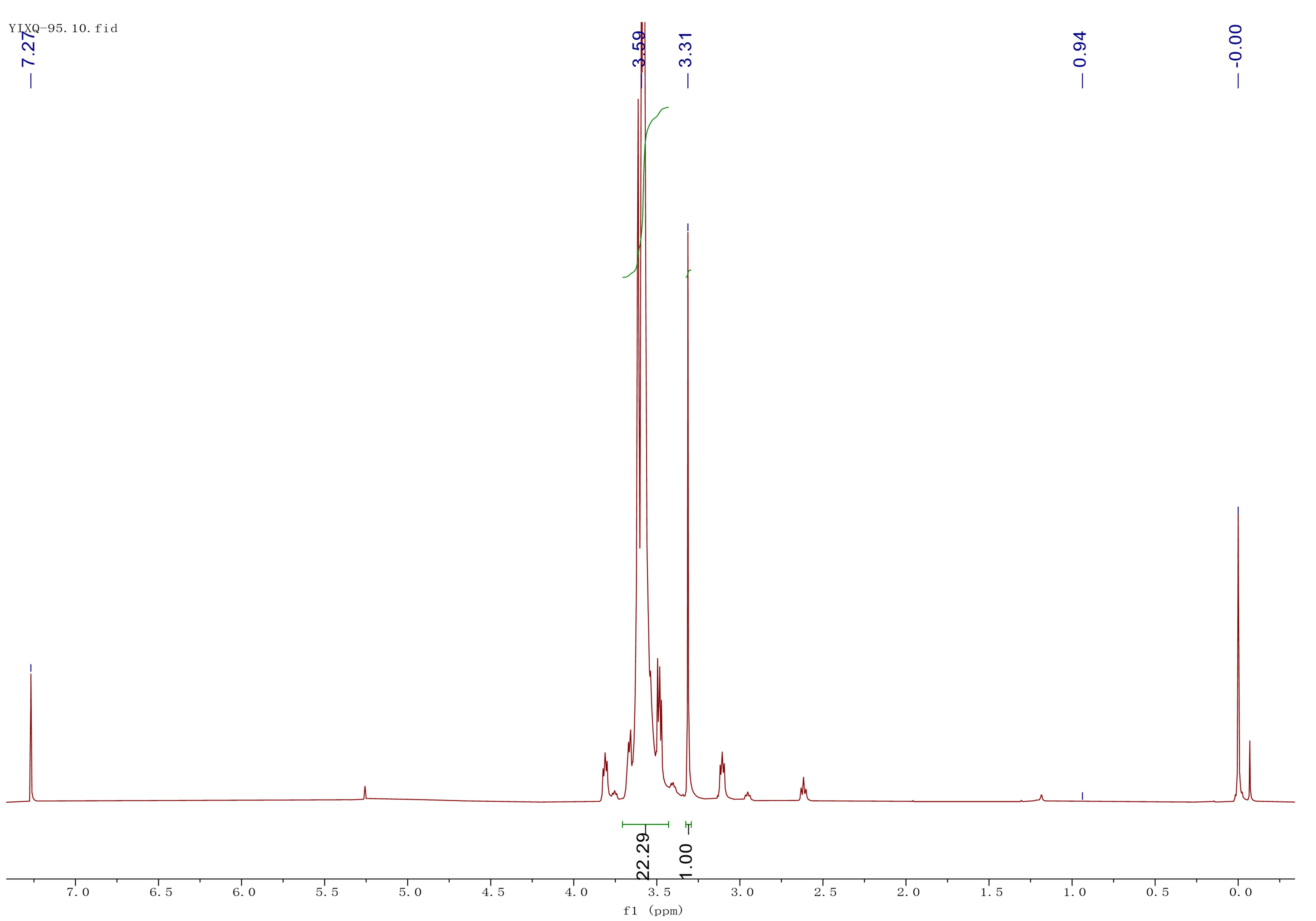


**Figure S3.** The ^1^H NMR spectrum (400 MHz, CDCl_3_) of mPEG-NH_2_.

_
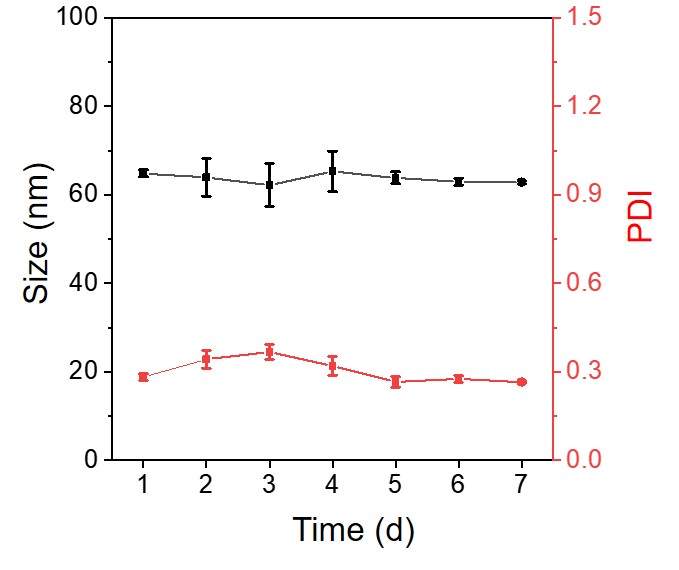
_

**Figure S4.** Seven-day size change of Ce6@DP.

_
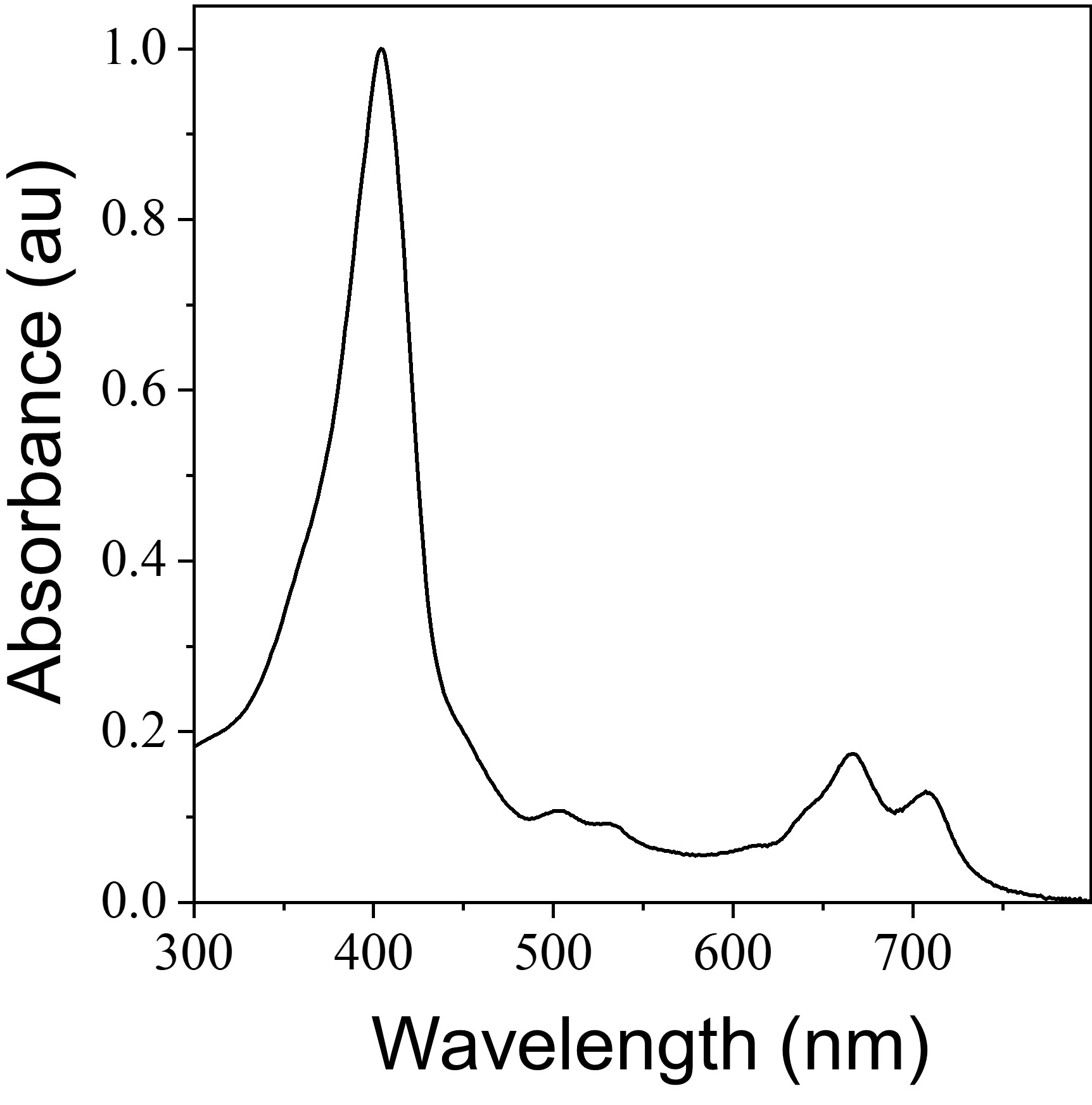
_

**Figure S5.** UV-Vis spectrum of Ce6@PTP/DP micelles in aqueous solution.


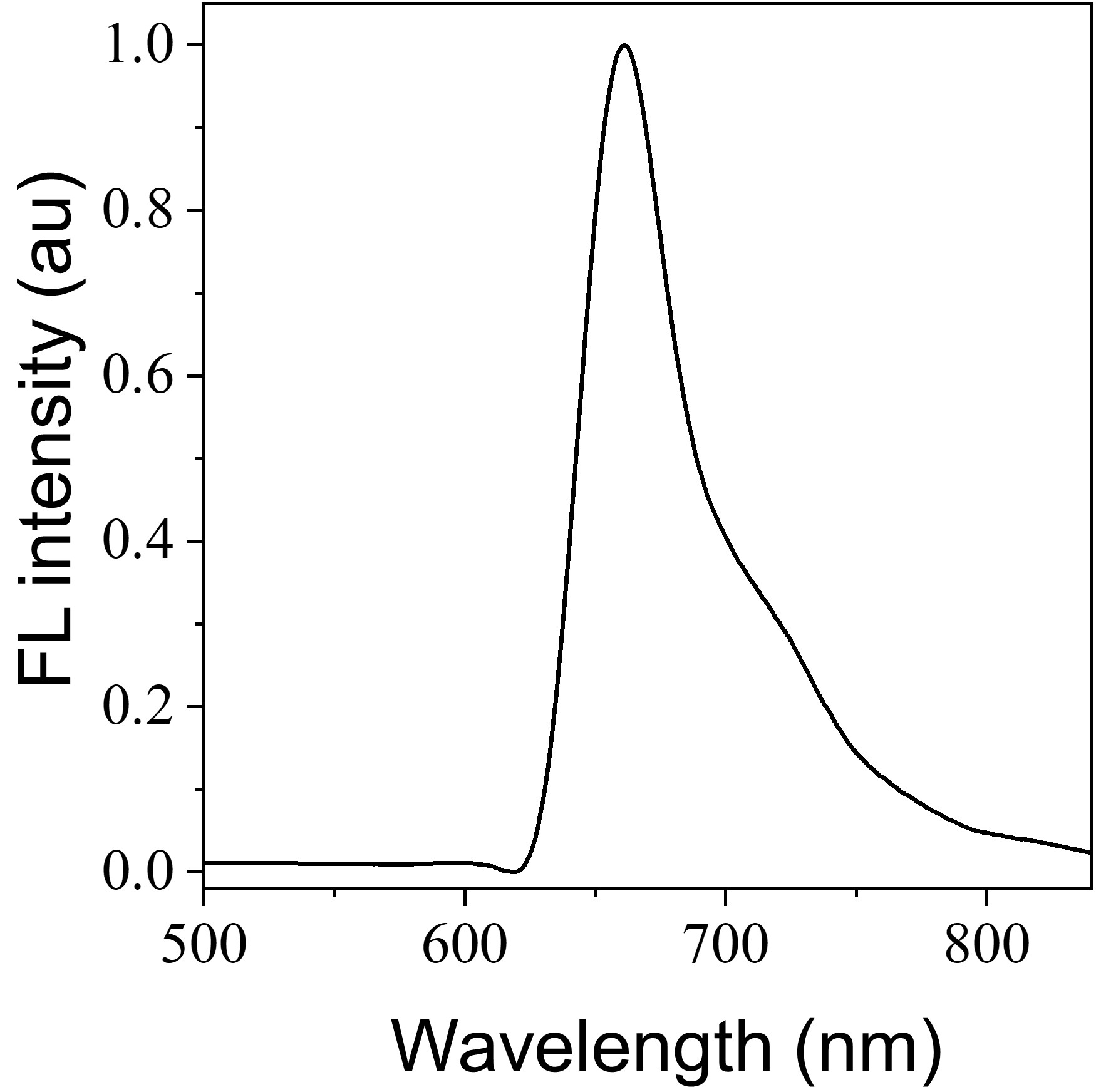


**Figure S6.** Fluorescence spectrum (FL) (Ex:400 nm) of Ce6@PTP/DP micelles in aqueous solution.


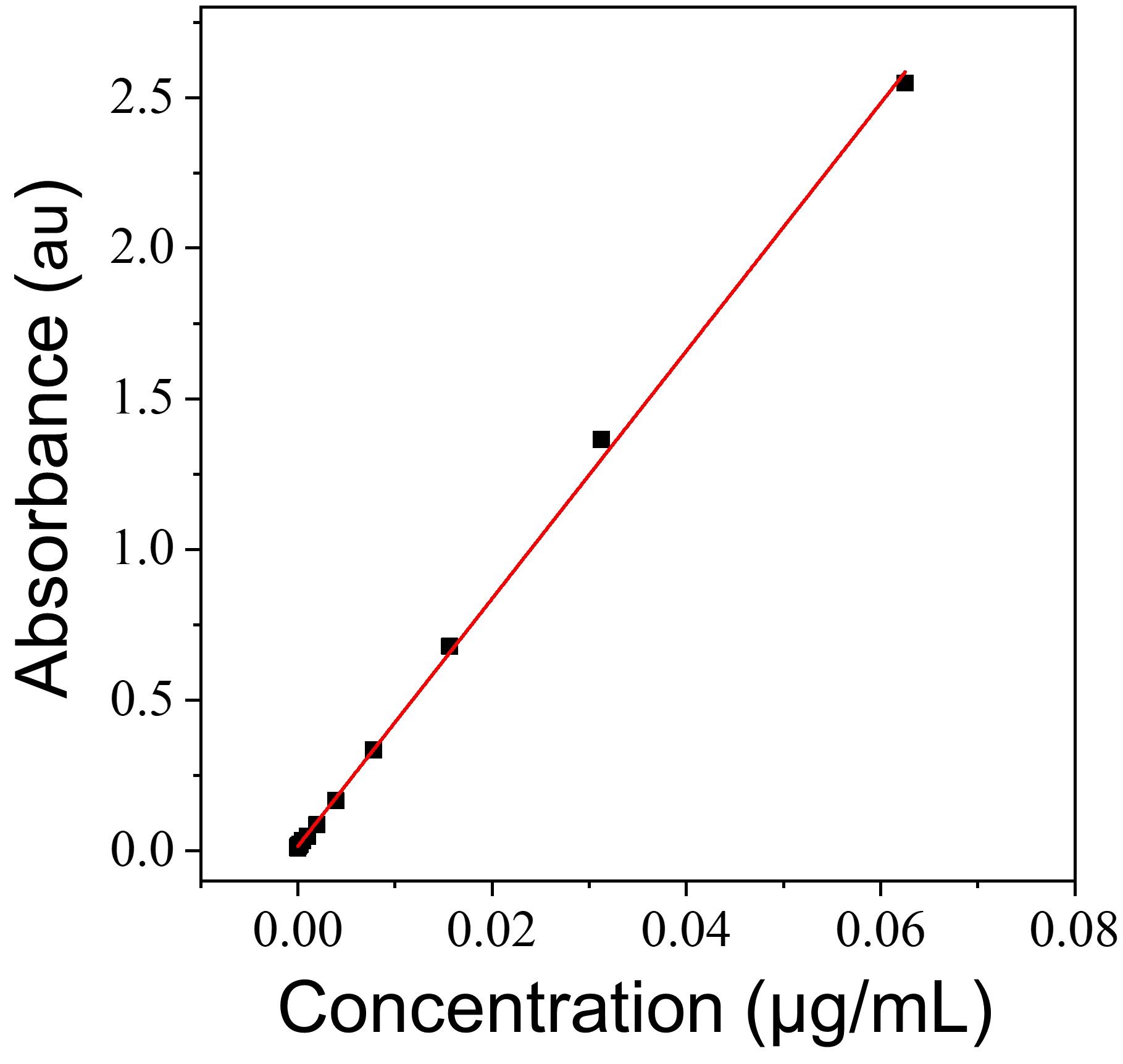


**Figure** **S7.** Standard curve of UV-Vis absorption of Ce6 in DMSO.


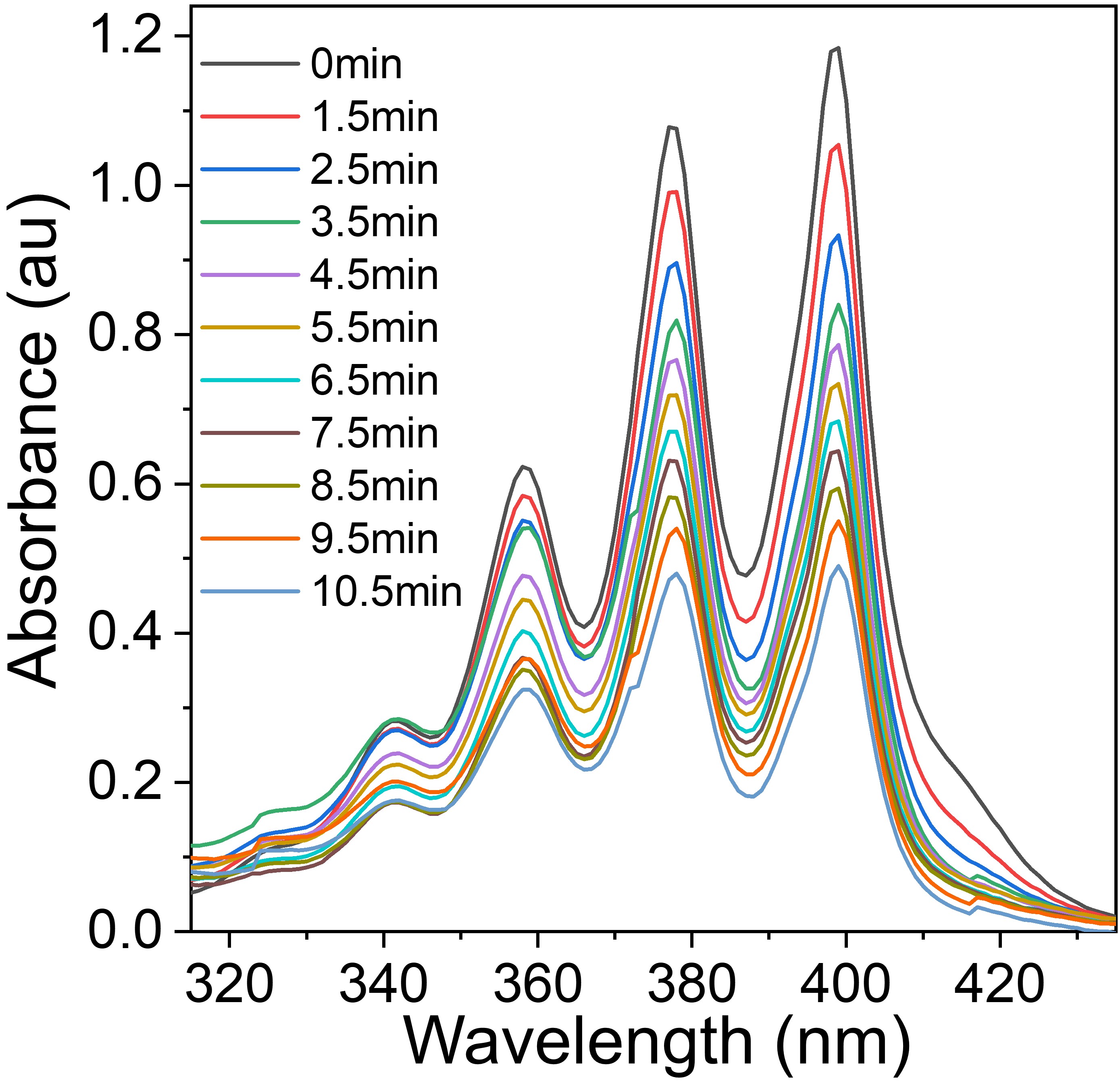


**Figure S8.** UV-Vis absorption spectra of the ROS indicator 9,10-anthracenediylbis (methylene) dimalonic acid (ABDA) mixed with Ce6@PTP/DP micelles upon light irradiation (white light, 100 mW cm^−2^).


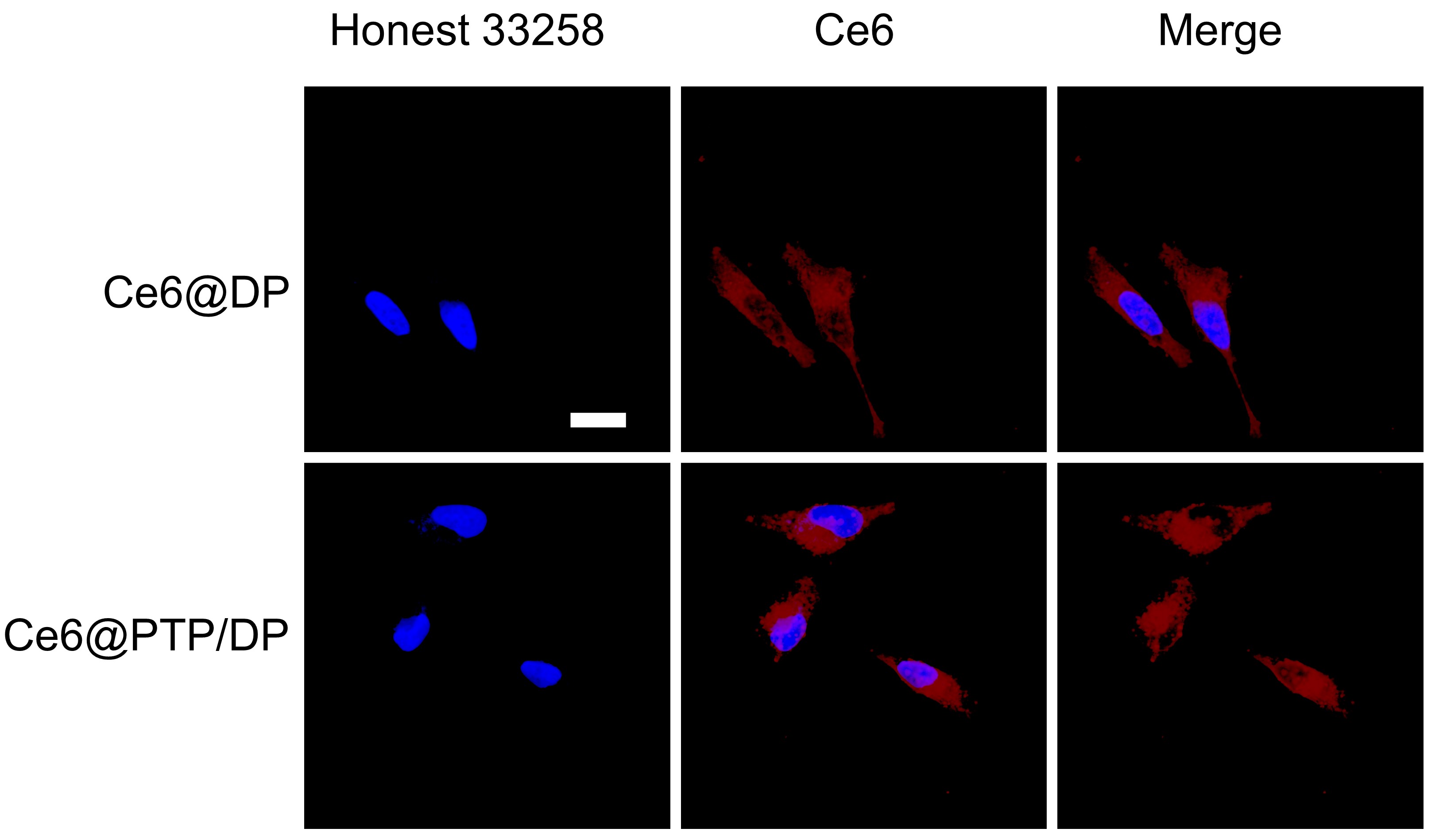


**Figure S****9.** Confocal images of HeLa cells after incubation with Ce6@DP and Ce6@PTP/DP micelles for 4 h, respectively. Scale bar: 20μm.


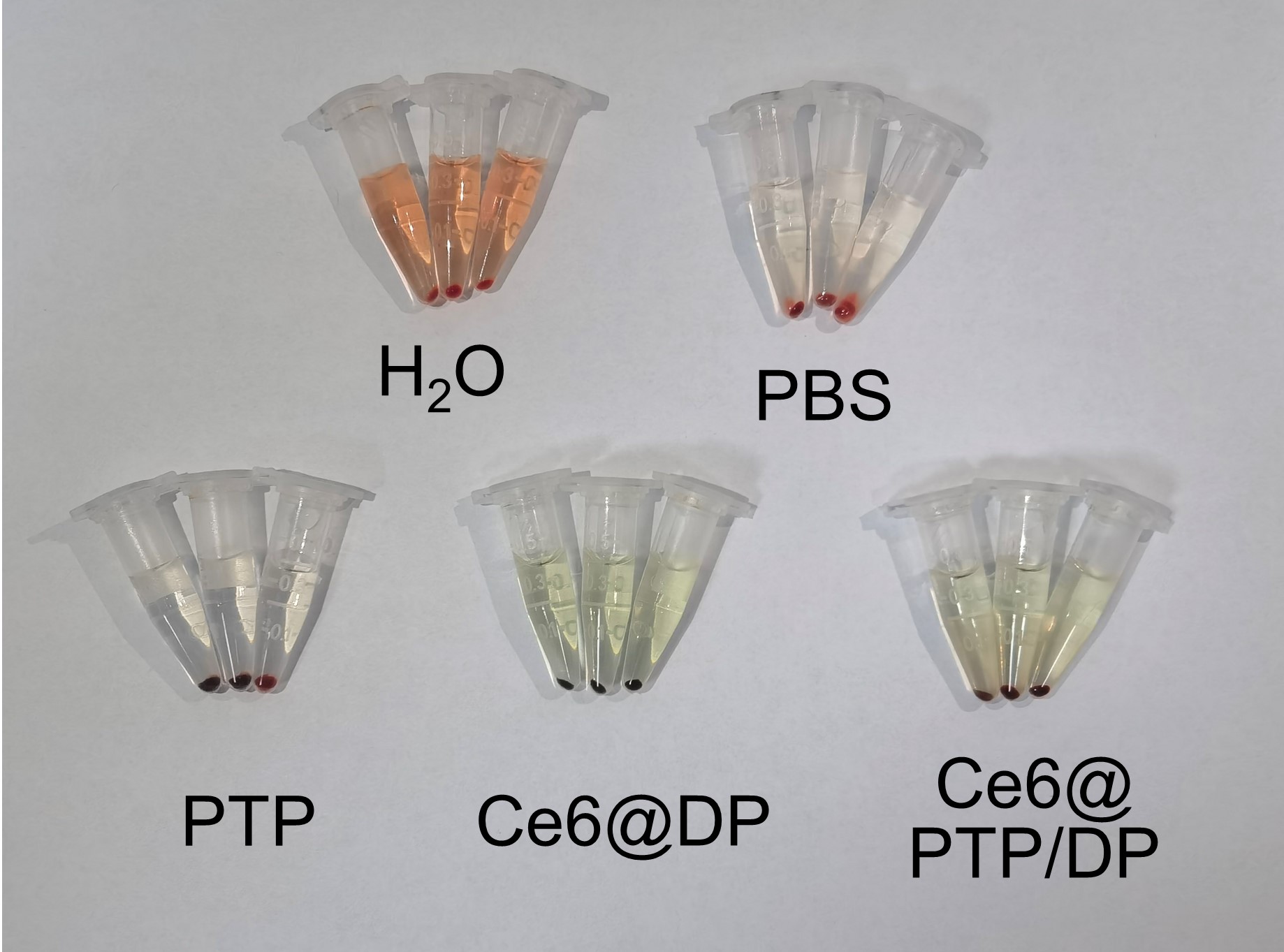


**Figure S10.** Photograph of hemolysis assay after incubation with ultrapure water (positive control), PBS (negative control), and PTP, Ce6@DP, Ce6@PTP/DP micelles for 6 h in dark, respectively.


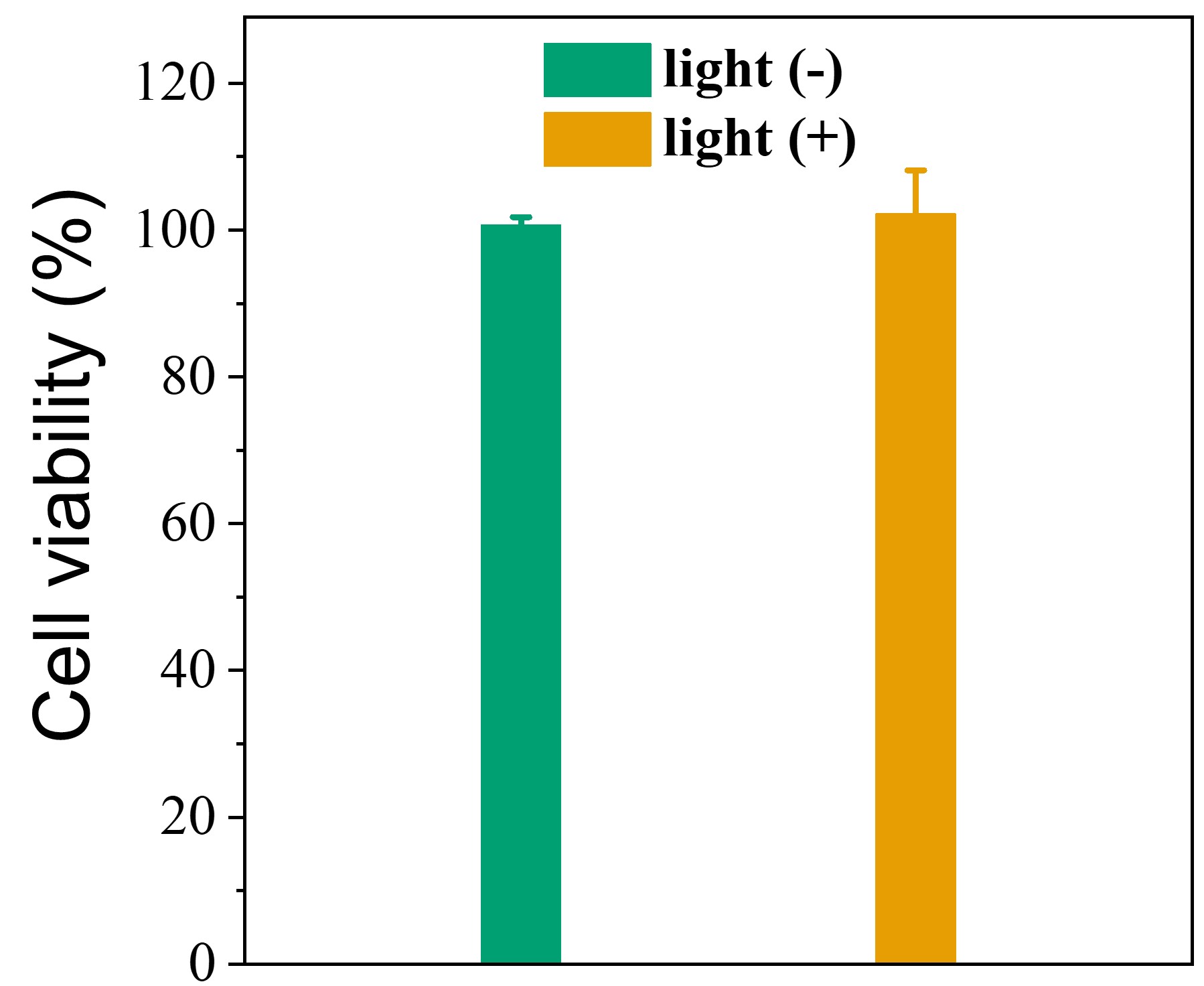


**Figure S11.** MTT assay of HeLa cells (with (+) and without (-) white light (100 mW cm^-2^)) incubated for 48 h. Data represent the mean ± s.d (n=3), respectively.


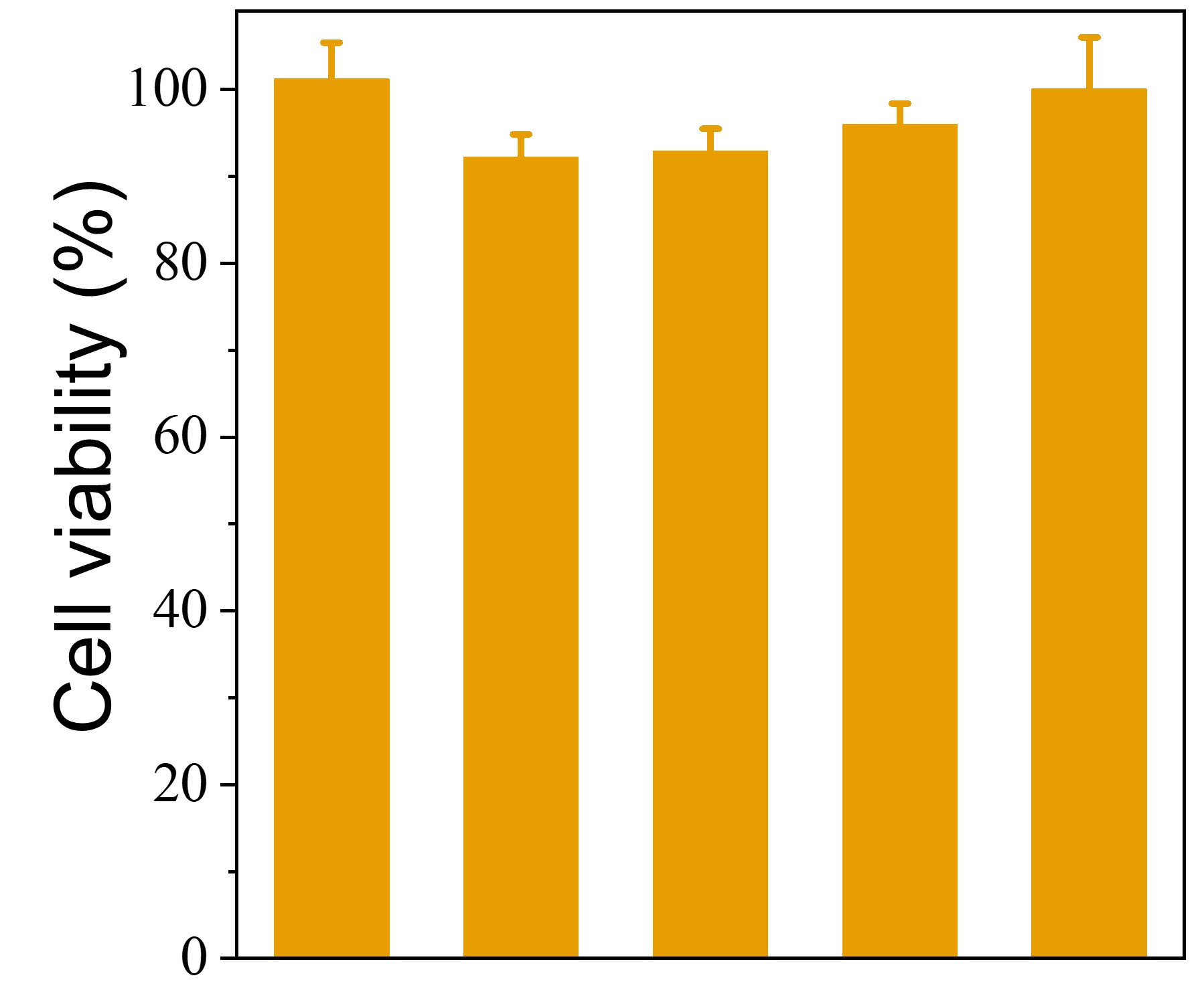


**Figure S12.** MTT assay of Ce6@DP micelles in HeLa cells after incubation for 48 h. Data represent the mean ± s.d (n=3).


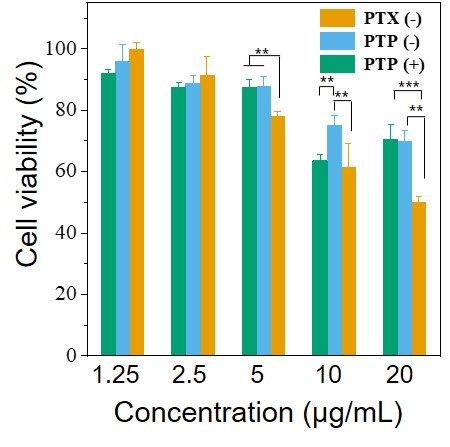


**Figure S13.** MTT assay of free PTX (-), PTP (-) and PTP (+) (white light, 100 mW cm^−2^, 10 min) in HeLa cells after incubation for 48 h. Data represent the mean ± s.d (n=3), **p* < 0.05, ***p* < 0.01, ****p* < 0.001.
